# Nursing Educational Interventions for Oncohaematological Patients Undergoing Autologous Stem Cell Transplantation and Their Caregivers: A Mixed-Methods Systematic Review

**DOI:** 10.3390/healthcare14213693

**Published:** 2026-11-01

**Authors:** Simona Sgromo, Fabrizio Petrone, Francesco Marchesi, Marta Cardone, Martina Siciliano, Antonella Volpe, Alessandro Spano, Giulia Manzon, Chiara Giammaria, Emanuele Di Simone, Nicolò Panattoni, Aurora De Leo

**Affiliations:** 1 Nursing Research Unit IFO, IRCCS Regina Elena National Cancer Institute, 00144 Rome, Italy; simona.sgromo@ifo.it (S.S.); fabrizio.petrone@ifo.it (F.P.); marta.cardone@ifo.it (M.C.); antonella.volpe.15@alumni.uniroma2.eu (A.V.); giulia.manzon@ifo.it (G.M.); chiara.giammaria@ifo.it (C.G.); aurora.deleo@ifo.it (A.D.L.); 2 Department of Biomedicine and Prevention, University of Rome Tor Vergata, 00133 Rome, Italy; martinasiciliano17@gmail.com (M.S.); alessandro.spano@responsible.hospital (A.S.); 3 Clinical and Experimental Oncohematology Unit, IRCCS Regina Elena National Cancer Institute, 00144 Rome, Italy; francesco.marchesi@ifo.it; 4 Responsible Research Hospital, 86100 Campobasso, Italy; 5 Department of Medical, Movement and Wellbeing Sciences, Parthenope University of Naples, 80133 Naples, Italy; emanuele.disimone@uniparthenope.it

**Keywords:** caregiver education, hematologic neoplasms, hematopoietic stem cell transplantation, mixed-methods systematic review, oncology nursing, patient education as topic

## Abstract

**Background/Objectives:** Autologous Stem Cell Transplantation (ASCT) has substantial physical and psychological implications for patients and caregivers. Although nurses play a crucial role in providing education throughout this journey, evidence on nursing educational interventions in this context remains limited. This Mixed-Methods Systematic Review aimed to synthesise their characteristics, outcomes, and associated experiences among adult patients undergoing ASCT and their caregivers. **Methods:** Following JBI convergent segregated methodology, we included quantitative, qualitative, and mixed-methods studies of nursing educational interventions for adults undergoing or preparing for ASCT and their caregivers. Studies explicitly involving paediatric or allogeneic transplant populations, as well as non-nursing interventions, were excluded. PubMed, Scopus, CINAHL, PsycINFO, and CENTRAL were searched from inception to 6 April 2026. Methodological quality was assessed using the Mixed Methods Appraisal Tool. Quantitative findings were synthesised narratively and qualitative findings through meta-aggregation. The protocol was registered in PROSPERO (CRD420251006310). **Results:** Seven reports representing six studies were included. One non-randomized study (60 participants) provided usable quantitative outcome data, reporting improvements in self-care and self-efficacy. Qualitative findings from two studies (55 participants) highlighted personalised information, emotional readiness, caregiver involvement, and peer support. Only one report directly included caregivers; no quantitative caregiver-reported outcomes were available. **Conclusions:** The limited quantitative evidence, with some uncertainty regarding its applicability, precluded firm conclusions about intervention effectiveness. However, qualitative evidence identified educational characteristics relevant to patients’ and caregivers’ experiences. Further rigorous research using validated patient- and caregiver-reported outcome and experience measures is needed. This work received funding from the Italian Ministry of Health.

## 1. Introduction

Haematological malignancies are neoplastic disorders that arise from alterations in normal blood cell production [1]. They are generally grouped into four main categories: leukemias, multiple myelomas, Hodgkin lymphomas, and non-Hodgkin lymphomas [2]. Together, according to the Global Cancer Observatory, these malignancies accounted for approximately 6.5% of all newly diagnosed cancers worldwide in 2024, with non-Hodgkin lymphoma representing the most frequently diagnosed subtype [3].

Although therapeutic advances in recent years have significantly improved the management of these malignancies [4], Autologous Stem Cell Transplantation (ASCT) remains a well-established standard of care [5,6]. Its use has been shown to improve patient survival across various clinical settings [7,8,9]. ASCT consists of collecting patients’ haematopoietic stem cells, storing them through cryopreservation, and reinfusing them after the administration of high-dose chemotherapy, with or without radiotherapy, aimed at eliminating residual tumour cells [10].

Despite its clinical effectiveness, ASCT can have a significant physical and psychological impact on both patients and their families [11,12]. For patients, major challenges include prolonged hospitalization, sleep disturbances, fear of death, changes in self-esteem, disruption of social relationships and daily activities, and several side effects such as nausea, vomiting, oral mucositis, haemorrhagic cystitis, neutropenia, and an increased risk of infections or sepsis [13,14,15,16,17]. Caregivers, in turn, often experience high levels of psychological distress and role burden, especially during the early stages of the transplant process [18,19]. Moreover, the unpredictable trajectory of the transplant process often makes it difficult for caregivers to prepare adequately for their evolving responsibilities [20]. In this context, Therapeutic Education (TE) emerges as a key strategy to support patients and caregivers in managing treatment-related side effects and adapting to the complexity of care [21,22]. TE is a structured process, led by qualified healthcare professionals, designed to strengthen people’s ability to self-manage their condition by mobilizing personal resources and actively involving caregivers [23]. Nurses, in particular, are identified by the Oncology Nursing Society as key providers of patient education and symptom management throughout the cancer care continuum [24].

Thanks to their constant presence, their ability to translate medical information into practical advice, and their close relationships with patients and families, nurses are ideally placed to support both self-care and emotional adjustment throughout the transplantation process.

However, despite the recognized value of TE and the central role of nurses in its delivery, patients undergoing or preparing for ASCT, as well as their caregivers, frequently report inadequate preparation regarding the complexity and progression of care [25,26].

While the need for more structured educational support is widely acknowledged [27], the literature still includes only a small number of studies evaluating such interventions [28]. Furthermore, these interventions are frequently delivered using heterogeneous methodologies, while evidence evaluating their outcomes remains limited, making it difficult to determine which educational approaches are most beneficial [29]. Experience from similar settings, such as allogeneic stem cell transplantation, suggests that structured educational programs can positively influence patients’ quality of life [30,31]. This evidence reinforces the importance of addressing the gap identified in the current literature.

The use of Patient-Reported Outcome Measures (PROMs), Caregiver-Reported Outcome Measures (CROMs), Patient-Reported Experience Measures (PREMs), and Caregiver-Reported Experience Measures (CREMs) when evaluating structured educational interventions may provide a more comprehensive and person-centred understanding of their impact [32,33]. PROMs and PREMs allow patients to directly report their symptoms, emotional well-being, disease-related knowledge, and quality of life—domains identified by the World Health Organization as core targets of TE [23]. CROMs and CREMs complement these measures by providing caregiver insights into readiness, stress levels, and practical caregiving capacity [32].

During a preliminary search conducted in PubMed and Scopus databases, as well as in the PROSPERO register, one review relevant to this topic was identified [29]. However, it included only randomized controlled trials involving mixed autologous/allogeneic populations, including post-transplant survivors, without specifically focusing on educational interventions involving nurses in their delivery. Furthermore, caregivers were excluded as a study population, and the review primarily examined intervention effectiveness in terms of quality of life, psychological distress, and patient satisfaction, without incorporating qualitative evidence on patients’ and caregivers’ experiences. These differences highlight the need for a more focused understanding of nursing educational interventions within the ASCT pathway, considering both their outcomes and their relevance to patients’ and caregivers’ needs. Given the lack of specific focus on educational interventions involving nurses in the existing literature, a systematic review was conducted to address this gap and support the future development of structured educational interventions in this context. The review focused on nursing interventions, as these represent an established component of educational strategies implemented in clinical practice. Recognizing the multifaceted needs of both patients and caregivers, as well as the importance of aligning intervention design with their perspectives, a Mixed-Methods Systematic Review [34] was conducted. This approach allows quantitative evidence on intervention outcomes to be considered alongside qualitative evidence on patients’ and caregivers’ experiences, values, and preferences, thereby providing a broader understanding of both intervention outcomes and the experiential dimensions of the educational process. This Mixed-Methods Systematic Review therefore aims to synthesise evidence on the characteristics, outcomes, and perceptions of nursing educational interventions for adult oncohaematological patients undergoing ASCT and their caregivers.

## 2. Aim

This Mixed-Methods Systematic Review aims to address the following research questions:

1. *Descriptive question (general scope)*: What types of nursing educational interventions are used for adult oncohaematological patients (≥18 years) undergoing or preparing for ASCT and their caregivers?
2. *Quantitative question:* What is the impact of nursing educational interventions on PROMs, CROMs, PREMs, and CREMs among adult oncohaematological patients (≥18 years) undergoing or preparing for ASCT and their caregivers?
3. *Qualitative question:* How do adult oncohaematological patients (≥18 years) undergoing or preparing for ASCT and their caregivers perceive nursing educational interventions?

## 3. Materials and Methods

### 3.1. Study Design

A Mixed-Methods Systematic Review was conducted in accordance with the Joanna Briggs Institute (JBI) Manual for Evidence Synthesis, following the convergent segregated approach [34]. The review was reported in line with the Preferred Reporting Items for Systematic Reviews and Meta-Analyses (PRISMA) guidelines [35]. The protocol was registered in the PROSPERO database of the National Institute for Health and Care Research (PROSPERO identification number: CRD420251006310), and no amendments were made after registration.

### 3.2. Search Strategy

The literature search strategy was developed to identify studies published in peer-reviewed journals and was based on the PICO (Population, Intervention, Comparison, Outcome) and PEO (Population, Exposure, Outcome) frameworks. An initial PubMed search was conducted in collaboration with a medical librarian to identify relevant index terms and free-text keywords. This search strategy was then adapted for use in all the other databases. The literature search was conducted across five electronic databases: PubMed, Scopus, CINAHL, PsycINFO, and CENTRAL (Cochrane Library). Searches were conducted from database inception to 6 April 2026. No search filters were applied. Only studies published in English or Italian were considered eligible. The complete search strategies are presented in Supplementary Table S1.

The reference lists from both included studies and excluded but relevant secondary sources were screened to identify any additional eligible studies.

### 3.3. Inclusion and Exclusion Criteria

#### 3.3.1. Population

This Mixed-Methods Systematic Review considered studies involving adult patients (≥18 years) diagnosed with haematological malignancies undergoing or preparing for ASCT. Studies involving conditions such as light-chain Amyloidosis and Polyneuropathy–Organomegaly–Endocrinopathy–Monoclonal gammopathy–Skin changes (POEMS) syndrome were also included, as these are considered pre-malignant or paraneoplastic disorders with therapeutic similarities to haematological malignancies, including the use of ASCT in selected cases [36,37].

In addition to patients, studies involving their informal caregivers were also included. Informal caregivers were defined as adult family members or close friends (aged ≥ 18 years) who provided assistance in a non-professional and unpaid capacity. Studies were excluded if they involved paediatric populations, adult caregivers of paediatric patients, or formal caregivers (paid professionals who provide care and assistance as part of their professional role).

#### 3.3.2. Intervention

The quantitative component of this review considered studies evaluating the impact of educational interventions delivered by at least one nurse. For the purposes of this review, nursing educational interventions were defined as interventions in which at least one nurse was actively involved in delivering educational content to patients and/or caregivers. Eligible interventions included both those delivered entirely by nurses and multidisciplinary interventions in which nurses contributed to one or more components of the educational process. Therefore, exclusive nursing responsibility for the intervention was not required. Multicomponent interventions were also eligible, provided that an educational component was clearly identifiable and at least one nurse was actively involved in its delivery. Studies reporting interventions conducted exclusively by healthcare professionals other than nurses (e.g., interventions limited to psychological or physiotherapy/rehabilitation approaches) were excluded. Studies that included a usual care group as a comparator were included, as well as those without a comparator.

#### 3.3.3. Phenomena of Interest

The qualitative component of this review considered studies that explored the experiences and perceptions of patients with haematological malignancies and their caregivers regarding structured nursing educational interventions delivered before or during the ASCT procedure. Studies focusing exclusively on perceptions of psychological or physiotherapy/rehabilitation-only interventions were excluded.

#### 3.3.4. Outcomes

The quantitative component of this review considered studies that assessed the impact of nursing educational interventions using PROMs, CROMs, PREMs, and CREMs. For the quantitative outcome synthesis, only results obtained using measurement instruments with documented evidence of validity or reliability, either reported in the primary studies or available from previous validation studies, were considered. Studies that did not meet this requirement were retained in the descriptive synthesis of educational intervention characteristics, provided that the other eligibility criteria were satisfied.

#### 3.3.5. Context

This review considered studies conducted in hospital and home settings, where nursing educational interventions were delivered either in person or remotely, including through digital tools. Interventions could be provided before or during the ASCT process. No geographical or cultural restrictions were applied in order to capture a broad perspective on educational practices across different healthcare systems.

#### 3.3.6. Types of Studies

This review included primary quantitative, qualitative, and mixed-methods studies of all designs, encompassing both experimental and non-experimental research. Quantitative studies included randomized controlled trials (RCTs), non-randomized controlled trials, pre–post studies (with or without a comparison group), interrupted time-series studies, and observational or descriptive studies, including retrospective designs. Qualitative studies were eligible if they employed well-established methodologies such as phenomenology, grounded theory, or ethnography to explore participants’ experiences and perceptions. Mixed-methods studies were included only when data from the quantitative or qualitative components could be clearly extracted. Secondary studies, protocols, editorials, posters, and conference papers were excluded.

### 3.4. Study Selection

Following the search, all the identified citations were collated and uploaded into Zotero, version 7.0.15 (Digital Scholar, Falls Church, VA, USA) [38], and duplicates were removed. Two reviewers independently screened the remaining records by title and abstract, applying the predefined inclusion and exclusion criteria. Potentially eligible studies were retrieved in full text, and relevant information was recorded in a Word file to document the screening process. Two reviewers independently assessed the full-text articles against the inclusion criteria. As specified in the review protocol, studies were excluded if they explicitly referred to paediatric populations, allogeneic transplant populations, or strictly psychological or physiotherapy/rehabilitation interventions. When relevant information concerning study eligibility was unclear or outcome data were inconsistently reported, clarification was sought from the corresponding author of the relevant primary study. In the absence of a response, eligibility decisions were based on a comprehensive examination of all available information in the full-text publications and associated additional files, including transplant-related treatment details reported in tables and the content of the educational interventions. In particular, when the transplant type was not explicitly reported, eligibility was assessed using the available clinical and educational information to determine whether the study context was compatible with the target ASCT population. These cases were not considered definitive classifications; rather, their applicability to the review population was considered uncertain, and their findings were interpreted cautiously during the quantitative synthesis and subsequent integration, particularly because the review identified mixed transplant populations in the literature and recognised that autologous and allogeneic pathways differ in clinically relevant ways. Nursing involvement in the delivery of educational interventions was assessed based on the descriptions provided in the primary reports, including how the interventions were characterised by the authors and the professional roles explicitly reported. When multiple publications referred to the same study or intervention, reports providing distinct and relevant data for the review questions were retained and linked to the same underlying study. Additional reports that did not provide relevant new data were excluded. Any disagreements at any stage of the selection process were resolved through discussion between the reviewers or with the supervision of an external reviewer with expertise in the methodology.

### 3.5. Quality Assessment Procedures

The methodological quality of all included reports was independently appraised by two reviewers using the Mixed Methods Appraisal Tool (MMAT), version 2018 (McGill University, Montréal, Canada) [39]. MMAT was selected to enable a consistent appraisal and presentation of methodological quality across heterogeneous study designs within a single framework. The MMAT is specifically designed to assess the quality of empirical studies across five methodological categories: RCTs, non-randomized studies, quantitative descriptive studies, qualitative research, and mixed-methods studies. It includes two preliminary screening questions applicable to all study types, followed by five sets of design-specific criteria. As recommended by the authors of the MMAT, the methodological quality of the included studies was not summarized using an overall score [39]. Instead, each criterion was assessed and presented separately to provide a clearer and more nuanced picture of the study quality. Data were extracted from all included studies regardless of their methodological quality. The methodological appraisal findings were used to inform the interpretation of the evidence, with particular attention to study-specific limitations and their potential implications for the reliability of the reported findings. Any disagreements during the critical appraisal process were resolved through discussion between the reviewers or with the supervision of an external reviewer with expertise in the methodology.

### 3.6. Data Extraction

Quantitative and qualitative data were independently extracted by two reviewers using a data extraction template specifically developed for this review. For each included study, data were collected on the first author, year of publication, country, aims, methods, participants’ details, characteristics of the educational interventions (including timing, format, contents, and duration/frequency), PROMs, CROMs, PREMs, and CREMs employed, and the main results. For quantitative studies, means, standard deviations, and sample sizes were extracted when reported. For qualitative studies, findings aligned with the review objectives were extracted along with their supporting illustrations. Based on the analysis of observational data and participant quotes, each finding was assigned a level of credibility—unequivocal (U), credible (C), or not supported (NS) [40]. Any disagreements that arose during the process were resolved through discussion between the reviewers or with the supervision of an external reviewer with expertise in the methodology.

### 3.7. Data Synthesis and Integration

This review followed the JBI methodology for Mixed-Methods Systematic Reviews, adopting a convergent segregated approach [34]. Quantitative and qualitative evidence were synthesized separately and then integrated in the final stage of the analysis. As this review addressed three distinct questions, different subsets of studies contributed to each synthesis: qualitative studies informed the qualitative synthesis, quantitative studies informed the quantitative synthesis, and all included studies contributed to the overall descriptive synthesis. A meta-analysis was planned when sufficient homogeneity and data were available; otherwise, quantitative findings were to be summarized narratively. Heterogeneity was explored descriptively based on differences in study characteristics, and no standardized effect metrics were applied due to variability in outcome reporting. As specified in the registered PROSPERO protocol, publication bias was planned to be assessed using funnel plot asymmetry and Egger’s or Harbord’s tests, where applicable. Qualitative synthesis was performed by the use of the meta-aggregation approach [40,41]. Findings assessed as unequivocal or credible were grouped into categories according to similarities in meaning. These categories were subsequently combined into a set of synthesized findings designed to support evidence-based practice. During the qualitative synthesis process, several informed decisions were made in order to balance methodological rigor with fidelity to the meanings reported by the primary authors. Consistent with JBI methodology [34], findings were extracted at either the theme or sub-theme level, depending on how clearly and directly the data addressed the review question. This approach was intended to ensure that the underlying meanings were fully captured [40]. When a finding was relevant to more than one category, it was reasonably assigned to multiple categories in order to avoid oversimplification and preserve conceptual richness [40]. When multiple reports originated from the same underlying study, the findings were examined to identify potential overlap in the reported themes and supporting quotations. Reports presenting distinct analytical perspectives and findings were retained in the qualitative synthesis to preserve their complementary contributions, while being treated as a single study rather than as independent sources of evidence. Finally, quantitative and qualitative findings were integrated through a narrative configured analysis, in accordance with JBI guidance for convergent segregated mixed-methods systematic reviews [34]. This involved comparing the findings from the two evidence streams to identify areas of convergence, complementarity, or contradiction; considering whether qualitative findings could provide contextual explanations for quantitative outcomes; examining differences in the direction and magnitude of quantitative effects where applicable; and identifying aspects addressed in one evidence stream but not explored in the other. Given the limited quantitative evidence, the integration focused primarily on identifying complementary findings, areas not addressed by both evidence streams, and implications for future research.

In accordance with the JBI Manual for Evidence Synthesis [34], no formal assessment of the certainty of evidence using GRADE or ConQual was planned. This methodological decision was consistent with current JBI guidance, which does not recommend applying these approaches to mixed-methods systematic reviews adopting either a convergent segregated or convergent integrated approach, given the complexities associated with evidence integration and certainty assessment.

### 3.8. Ethical Consideration

As this study is a systematic review of previously published research, no primary data were collected; therefore, ethical approval was not required. According to the primary authors, all included studies were conducted in accordance with established ethical standards. In our synthesis, particular care was taken to represent participants’ perspectives accurately and respectfully, especially when integrating qualitative findings.

## 4. Results

**Figure 1:**
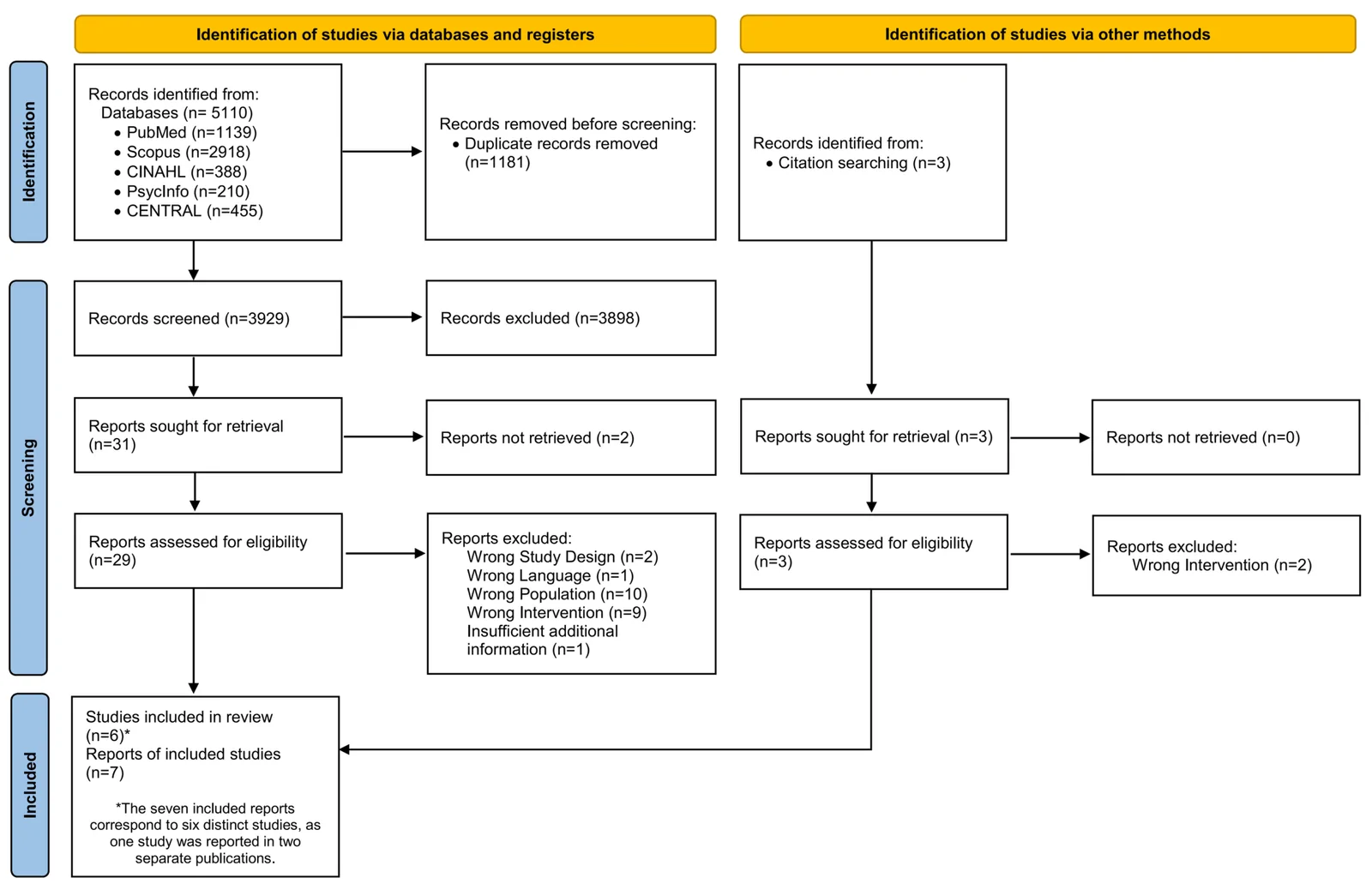
PRISMA Flow Diagram of the study selection process.

As illustrated in Figure 1, following duplicate removal and title–abstract screening, thirty-two full-text reports were retrieved and assessed for eligibility: twenty-nine were identified through database searching and three through citation searching. Reports excluded at this stage of the screening process are listed in Supplementary File S1.

A total of seven reports, representing six unique studies, met the inclusion criteria and were included in the review. All seven reports contributed to the descriptive question, whereas only one report contributed to the quantitative question and three reports contributed to the qualitative question. All seven reports underwent methodological quality appraisal.

### 4.1. Quality Appraisal

As shown in Table 1, all seven reports met the two initial screening questions, allowing them to proceed to the design-specific appraisal. While the reports contributing qualitative evidence [42,43,44] met all methodological criteria, some limitations were identified among the reports contributing quantitative evidence, mainly related to incomplete methodological reporting. In particular, three reports had reporting limitations: two did not provide information regarding dropouts or missing questionnaire data [45,46], and one did not report the validation or reliability testing of the measurement instruments used [47]. Issues related to sample adequacy were identified in two reports. One reported an 18% dropout rate, with 11% of participants being too emotionally distressed or physically debilitated to complete the intervention [47], while another included a small sample (*n* = 10) [45]. In addition, one report described an observational design to evaluate intervention effects, potentially limiting the ability to account for confounding factors [48]. The full MMAT criteria applicable to each included report, together with the corresponding judgments and explanations for ratings of “No” or “Can’t tell”, are provided in Supplementary Table S2.

### 4.2. Main Characteristics of Included Studies

The characteristics of the seven reports, representing six unique studies, included in the descriptive synthesis are summarized in Table 2, together with the characteristics of the educational interventions and their reported findings. Overall, the reports were published between 1999 and 2026 and originated from studies conducted in different countries: three in the United States [42,43,44], one in Canada [45], one in the Netherlands [47], and two in China [46,48]. The included reports comprised four quantitative [45,46,47,48] and three qualitative investigations [42,43,44]. Among the quantitative reports, one described a non-randomized controlled trial [46], one used a one-group post-test-only design [47], one reported a quality improvement project [45], and one used a retrospective observational design comparing outcomes between two groups receiving different care protocols [48]. Among the qualitative reports, two adopted a phenomenological approach [43,44], whereas the other followed an ethnographic design, drawing on observations and semi-structured interviews [42]. Two publications [42,43] were derived from the same underlying study but addressed different objectives and reported distinct findings. The qualitative evidence explored participants’ perceptions and experiences of educational interventions at different stages of the educational process. 

Six reports focused exclusively on oncohaematological patients [43,44,45,46,47,48], whereas only one report included data from both patients and caregivers [42]. Overall, the impact of nursing educational interventions was primarily assessed through outcomes related to psychological well-being [46], satisfaction with the information received [47], treatment adherence [45,48], and clinical outcomes [45,48].

### 4.3. Descriptive Question: What Types of Nursing Educational Interventions Are Used for Adult Oncohaematological Patients (≥18 Years) Undergoing or Preparing for ASCT and Their Caregivers?

Nursing educational interventions were delivered using a variety of heterogeneous methods. Most interventions were delivered exclusively during the pre-transplant phase [42,43,44,46,47], whereas some interventions were delivered entirely during hospitalization for ASCT [45,48]. Even when educational interventions were provided exclusively during the pre-transplant phase, patients had the opportunity to revisit the educational content over time. More broadly, access to educational content was facilitated by the provision of printed materials, such as booklets, folders, or guides [42,43,44,45,46], the possibility of contacting the researchers for clarification [46], or access to digital resources for subsequent consultation [42,43,47,48]. When provided, digital resources complemented but did not replace the face-to-face encounter. These resources included video-based programs [42,43], operative video instructions [48], and interactive CD-ROMs combining videos, animations, and printable text [47].

In one study, digital material was provided to patients after the in-person session [47], whereas in another it was emailed to patients before the in-person session as preparatory material [42,43]. The educational interventions covered a range of topics, from general transplant education [42,43,44,47] to more specific content, including oral care [45,48], stress-reduction strategies, and practical self-care instructions [46]. In one study, patients also received information about the hospital environment, including a description of the inpatient ward and an introduction to staff, as well as information about patient support organizations [47]. All educational interventions followed structured teaching protocols. In two reports, the authors described adapting the educational content to individual patient needs [42,44]. Where reported, intervention duration ranged from one-hour video sessions to 90-min in-person meetings [42,43].

### 4.4. Quantitative Question: What Is the Impact of Nursing Educational Interventions on PROMs, CROMs, PREMs, and CREMs in Adult Oncohaematological Patients (≥18 Years) Undergoing or Preparing for ASCT and Their Caregivers?

Three reports assessed nursing educational interventions using validated patient-reported outcome measures. Two reports originating from the same study [42,43] reported the use of the seven-item Functional Assessment of Cancer Therapy–General (FACT-G7) questionnaire [49], while the third report assessed self-care and self-efficacy using the Strategies Used by People to Promote Health (SUPPH) questionnaire [46,50]. However, the reports employing the FACT-G7 did not provide quantitative outcome data. Consequently, only one report provided usable quantitative outcome data for the synthesis, which was therefore limited to self-care and self-efficacy. No quantitative caregiver-reported outcome data were available. The only report contributing usable quantitative outcome data was considered plausibly relevant to the target ASCT population on the basis of the contextual information available in the primary report; however, as the transplant type was not reported with complete clarity, these findings should be interpreted cautiously, given their uncertain applicability to the review population. Given that only one report provided usable quantitative outcome data, the planned statistical assessment of publication bias, including funnel plot asymmetry assessment and Egger’s or Harbord’s tests, could not be performed.

#### Self-Care and Self-Efficacy

Wang et al. [46] investigated self-care and self-efficacy outcomes in a non-randomized controlled trial involving 60 participants. Initially, participants were assigned to either the intervention or control group by lottery. The remaining participants were then allocated to each group based on gender, until both groups had a total of 30 participants. The two groups were comparable in terms of age (41.2 ± 12.86 years in the intervention group; 44.46 ± 9.75 years in the control group), gender (50% male in both groups), educational qualifications (50% of participants in the intervention group and 43.3% in the control group had a diploma or university degree), economic status (40% in the intervention group and 46.7% in the control group had low economic status), and number of previous hospitalizations (40% had been hospitalized two to three times in both groups). No data were provided regarding participants’ diagnoses. Although the study was retained in the review because the available contextual elements made its inclusion in the target ASCT population plausible, the transplant type was not explicitly characterised in sufficient detail in the primary report. This aspect should therefore be considered when interpreting the findings. Prior to transplantation, the intervention group received an individualized, face-to-face educational session focusing on health promotion strategies. Topics included stress reduction, decision-making, adaptability, daily activities, rest, nutrition, and medication use. Each participant also received a printed booklet and had the opportunity to contact the researchers by phone for additional support. Although the intervention was described as a “nursing intervention” in the title of the primary publication, the professional qualification of the research collaborator who delivered the education was not explicitly reported. The control group continued to receive routine care provided by the transplant unit. Baseline data were collected before the educational sessions and outcomes were reassessed 30 days after transplantation using the SUPPH questionnaire (Cronbach’s α = 0.94). Within the intervention group, total self-efficacy scores increased from 81.36 ± 18.59 at baseline to 91.23 ± 14.62 at follow-up (*p* = 0.002). Statistically significant within-group improvements were also reported in the adaptability, decision-making, and stress-reduction subscales (all *p* = 0.001), whereas no significant change was observed in the enjoyment of life subscale (*p* = 0.160). No statistically significant within-group changes were reported in the control group. The authors also reported a statistically significant result from an analysis of covariance adjusted for potential confounders (*p* = 0.001), although the adjusted effect estimate and its confidence interval were not provided. Taken together with the limited reporting on population characteristics, the absence of an adjusted effect estimate and confidence interval further limits the interpretation of the reported intervention effect. Discrepancies were identified in the reporting of outcome data in the primary study, particularly regarding changes in self-efficacy scores. The corresponding author was contacted for clarification, but no response was received. Therefore, for the purposes of this review, we relied on the reported pre- and post-intervention mean scores, which indicated a smaller increase in total self-efficacy (+9.87 points) than the change reported elsewhere in the primary study (+14.83 points). In addition to the outcomes considered in this review, Wang et al. [46] reported a significant reduction in the expression of two anxiety-related genes, 5-HT1A and CRHR1, in the intervention group, as assessed by reverse transcription polymerase chain reaction (RT-PCR) (*p* = 0.01). These biological outcomes were not prespecified in the review protocol and fell outside the scope of the quantitative research question. They are therefore reported solely as exploratory observations from the primary study and were not included in the quantitative outcome synthesis.

### 4.5. Qualitative Question: How Do Adult Oncohaematological Patients (≥18 Years) Undergoing or Preparing for ASCT and Their Caregivers Perceive Nursing Educational Interventions?

Three reports representing two unique studies contributed to the qualitative synthesis [42,43,44], as two reports originated from the same underlying study [42,43]. From these reports, a total of 15 findings relevant to the research question were extracted. According to the JBI Manual for Evidence Synthesis [40], 12 findings were classified as Unequivocal, two as Credible, and one as Not Supported. As reported in Table 3, the 14 Unequivocal or Credible findings were grouped into four categories, which were subsequently merged into two Synthesised Findings.

#### 4.5.1. Synthesised Finding 1—Personalisation of Content and Emotional Management of Information Delivery Shape How Nursing Educational Interventions Are Experienced

This finding integrates two categories: “Need for personalised and balanced information” and “Emotional impact of educational interventions”. Patients frequently expressed a strong desire for information tailored to their individual needs and delivered progressively over time. Evidence directly collected from caregivers also highlighted difficulties in managing large amounts of educational information. Among patients, receiving substantial amounts of information at once was associated with feelings of being overwhelmed, confused, or distressed. In some cases, this led them to stop or avoid educational sessions, particularly when the focus shifted to complications or end-of-life topics:

“[…] as was the case of one 71-year-old white female who ended the visit early by stating, “This is just too much information...I’m done now—no more information”

[42] (p. 784).

By contrast, other participants reported feeling more reassured when given detailed explanations early:

“Find out everything in advance down to the most minute detail…. Become as close to an expert as you can without going to medical school”

[44] (p. 105).

Overall, the learning process itself was often described as emotionally intense. While some participants reported that it promoted a sense of preparedness and control, others associated it with anxiety, fear, or denial. The use of vague or understated language during educational sessions was also reported to cause confusion and, at times, mistrust, particularly when patients subsequently experienced symptoms that were more severe than anticipated. Interventions that allowed patients to revisit information, for example through videos or printed materials, or that provided realistic information in small, manageable steps were perceived as more helpful and less distressing.

#### 4.5.2. Synthesised Finding 2—Caregiver Involvement and Peer Support Are Perceived as Important Components of the Educational Process

This finding integrates two categories: “Peer interaction to reduce anxiety and uncertainty” and “The active involvement of caregivers in educational interventions”. The inclusion of caregivers in the educational process was considered important, not only because of their practical role in home care (e.g., medication management and central line care), but also because of the emotional support they provided. Patients also described caregiver education as beneficial, as it reduced the perceived burden of having to explain medical information to family members:

“Ana remembered how upset her sister was when a nurse told her not to visit Ana at a time when Ana’s white blood cell count was dangerously low. Without understanding that this was routine procedure, Ana’s sister assumed that something was terribly wrong”

[44] (p. 107).

Evidence concerning caregiver involvement emerged from both patients’ accounts and caregivers’ direct experiences, although the latter were available from only one included report [42]. In particular, one caregiver described difficulties managing the volume of written educational information and identifying the most essential content. Conversely, accessible educational resources, such as videos that could be reviewed repeatedly, were perceived as helpful in preparing for educational sessions and supporting caregiver participation.

For example, an ethnographic observation documented how a caregiver and patient had actively engaged with the educational materials and prepared questions ahead of their in-person session:

“Isabelle and John each took out the note sheets which were filled with questions they are written in previously blank spaces […] Ultimately, John and Isabelle stated they wanted to ask their questions later and Isabelle asked [Nurse] to do what she normally does, and they would ask the questions later.”

[42] (p. 783).

At the same time, interaction with patients who had already undergone ASCT emerged as an important source of emotional reassurance. In some cases, the experiences shared by fellow survivors were described as more reassuring than clinicians’ explanations, as lived experience was perceived as particularly meaningful alongside technical information:

“I think that when you go through a treatment, although it is explained, I think you really don’t know what you’re getting yourself into until you’re in the midst of it…. I think you have to go through it to really be able to know”

[44] (p. 107).

However, participants also emphasized the need to filter overly negative or unhelpful stories, highlighting the importance of carefully selecting which survivors to talk to.

### 4.6. Integration of Quantitative Evidence and Qualitative Evidence

The integration of quantitative and qualitative evidence was conducted through a narrative configured analysis, following the five questions proposed by the JBI Manual for Evidence Synthesis for mixed-methods systematic reviews adopting a convergent segregated approach [34]. The integration considered the relationships between the two evidence streams, their potential explanatory contributions, and the aspects addressed by one stream but not the other. Given the limited quantitative evidence base, the integration also considered the degree to which the quantitative findings could be regarded as directly applicable to the target ASCT population. In particular, the only report contributing usable quantitative outcome data was interpreted with caution, as its relevance to the review population was considered plausible on the basis of contextual elements, but could not be confirmed with complete certainty from the primary report.

#### 4.6.1. Convergence and Divergence Between Quantitative and Qualitative Findings

The quantitative and qualitative findings were primarily complementary, although direct convergence could not be established. Quantitative evidence from one report [46] described improvements in patients’ self-care and self-efficacy following an educational intervention combining face-to-face education, printed materials, and opportunities to contact healthcare staff for clarification. The qualitative synthesis [42,43,44] highlighted the importance of personalised information, progressive educational delivery, and opportunities to revisit educational materials. These findings were consistent with some characteristics of the intervention evaluated quantitatively, particularly the availability of supplementary educational resources and ongoing support. However, this complementarity should be interpreted cautiously, because it reflects a comparison between qualitative findings and a single quantitative report whose applicability to the target ASCT population was contextually supported but not fully verifiable from the published report. No direct contradictions were identified between the two evidence streams. Nevertheless, the absence of contradictory findings should not be interpreted as confirmation of agreement, given the limited quantitative evidence and the differences in outcomes and research questions addressed by the included studies.

#### 4.6.2. Contribution of Qualitative Evidence to Understanding Intervention Effectiveness

The qualitative evidence provided contextual insights into how nursing educational interventions were experienced by patients and caregivers. Participants described the importance of receiving information tailored to their needs, having opportunities to revisit educational materials, and receiving information progressively, with consideration of their emotional readiness. Conversely, receiving large amounts of information at once, without adequate attention to its emotional impact, was associated with feelings of overload, confusion, and anxiety. These findings suggest that the perceived value of educational interventions may depend not only on their informational content but also on how and when information is delivered. Although these experiences may help contextualise the quantitative findings reported by Wang et al. [46], they cannot establish why improvements in self-care and self-efficacy were observed. The qualitative and quantitative evidence originated from different studies, and the available data do not permit direct examination of the mechanisms underlying the reported outcomes.

#### 4.6.3. Explanation of Differences in the Direction and Magnitude of Quantitative Effects

The contribution of qualitative evidence to explaining differences in the direction or magnitude of intervention effects across quantitative studies could not be assessed. Although three reports used validated patient-reported outcome measures, only one provided usable quantitative outcome data [46]. Consequently, no comparisons of intervention effects across independent quantitative studies were possible. The qualitative evidence therefore could not be used to explain between-study variations in effect direction or magnitude.

#### 4.6.4. Quantitative Outcomes Not Explored in the Qualitative Evidence

The quantitative synthesis focused on patients’ self-care and self-efficacy, including adaptability, decision-making, stress reduction, and enjoyment of life [46]. Although the qualitative synthesis identified experiences related to emotional preparedness, perceived control, and information management, these findings did not constitute direct qualitative explorations of the quantitative outcomes measured in the study by Wang et al. [46]. Moreover, the interpretability of these quantitative outcomes is constrained not only by the lack of direct qualitative counterparts but also by the limited certainty regarding the study’s direct applicability to the review population.

#### 4.6.5. Qualitative Findings Not Assessed in the Quantitative Evidence

The qualitative synthesis identified several dimensions of educational experiences that were not directly assessed in the available quantitative evidence. These included the perceived importance of personalised and progressively delivered information, emotional readiness, opportunities to revisit educational materials, caregiver involvement, and peer support [42,43,44]. In particular, caregiver involvement emerged as an important aspect of the educational process, both in supporting patients and in addressing caregivers’ own informational and emotional needs. However, only one included report provided data directly collected from caregivers, and no quantitative caregiver-reported outcome data were available. Similarly, although peer support and the emotional consequences of educational communication were identified as relevant aspects of participants’ experiences, these dimensions were not directly evaluated as outcomes in the quantitative study.

## 5. Discussion

The primary objective of this Mixed-Methods Systematic Review was to describe the characteristics of nursing educational interventions delivered to adult oncohaematological patients undergoing or preparing for ASCT, as well as to their caregivers. The specific objectives were to assess the impact of these interventions on PROMs, CROMs, PREMs, and CREMs, and to explore how patients and caregivers perceive these interventions.

A key finding of this review is that the body of evidence specifically evaluating nursing educational interventions in the ASCT setting remains small and methodologically limited. Only seven reports met the inclusion criteria, highlighting the scarcity of research in this setting. While the quantitative evidence remains insufficient to establish intervention effectiveness, the qualitative findings provide preliminary insights into how educational interventions are experienced and which characteristics may be particularly relevant to patients’ and caregivers’ educational needs. From a quantitative perspective, only one included study provided usable outcome data, reporting significant improvements in patients’ self-care and self-efficacy [46]. However, these quantitative findings should be interpreted with caution, as the applicability of this single study to the target ASCT population was considered plausible on the basis of contextual elements but could not be confirmed with complete certainty from the primary report. The same study also reported changes in the expression of two anxiety-related genes (5-HT1A and CRHR1), although their clinical relevance remains uncertain [46].

An additional consideration concerns the heterogeneity of the interventions identified in this review. These differed not only in their educational content, delivery methods, and timing, but also in the extent to which education was integrated with other components of nursing care. This raises a broader methodological challenge regarding the extent to which the contribution of TE can be distinguished and evaluated within multicomponent interventions. For example, the intervention described by Wu et al. [48] combined patient education with nursing supervision, adherence monitoring, corrective feedback, and practical coaching. Although its inclusion was consistent with the review’s eligibility criteria, the observed clinical outcomes cannot be attributed specifically to the educational component. More broadly, when education is embedded within complex nursing interventions, determining its relative contribution to patient- and caregiver-related outcomes may be particularly challenging, as the different components are often closely interconnected and may influence one another. In this context, a more detailed description of educational activities and their relationship with other intervention components might offer a clearer understanding of how TE may contribute to patient- and caregiver-related outcomes. Similarly, methodological approaches aimed at examining both the contribution of individual components and their potential interactions might provide further insights into the role of TE within complex nursing interventions, although disentangling their respective effects may remain challenging.

Alongside the limited quantitative evidence, the qualitative synthesis offered preliminary insights into how nursing educational interventions were experienced by patients and caregivers. Across the two contributing studies, participants described educational experiences shaped not only by the information provided, but also by its timing, delivery, and responsiveness to individual circumstances. Personalisation of content, progressive information delivery, opportunities to revisit educational materials, and attention to emotional readiness emerged as important characteristics of the educational experience [42,43,44]. Conversely, receiving large amounts of information at once or without adequate consideration of its emotional impact was associated with anxiety, confusion, and feelings of being overwhelmed [42,44]. Although these findings derive from a small qualitative evidence base, they suggest that the perceived value of education may depend on relational and emotional aspects as well as informational content.

Related findings have been reported in previous research on educational interventions in hematopoietic stem cell transplantation, although differences in transplant populations and intervention characteristics should be acknowledged. For example, one study reported that supplementing standard verbal and written information with audiovisual materials improved patient and donor satisfaction, particularly in relation to understanding the written informed consent procedure and the potential risks of stem cell transplantation [51]. Furthermore, a study conducted in a mixed-transplant population reported that an educational program consisting of two face-to-face self-efficacy training sessions and a booklet produced a statistically significant increase in participants’ self-efficacy [52].

Beyond the outcomes examined in previous studies, the qualitative evidence from this review draws attention to the importance of aligning educational interventions with patients’ and caregivers’ lived experiences, emotional readiness, and evolving needs. Educational approaches that remain predominantly unidirectional and healthcare-professional-led may offer limited opportunities to incorporate these perspectives. Greater involvement of patients and caregivers in the development of educational interventions may therefore help ensure that educational strategies reflect not only clinical priorities but also the needs, preferences, and experiences of those directly involved in the transplant journey. In this regard, emerging frameworks such as co-design [53] and human-centred design [54] offer structured methodologies for systematically involving patients and caregivers in the ideation, development, and evaluation of healthcare interventions. These approaches emphasise iterative processes—including understanding users’ experiences, defining needs, prototyping, and testing solutions—that enable the co-creation of interventions grounded in real-world experiences rather than assumptions. Evidence from oncohaematological settings supports this perspective, with participatory and user-centred approaches being associated with greater relevance, usability, and acceptability of interventions, as well as patient empowerment and responsiveness to evolving needs [55,56]. Conversely, limited user involvement may hinder intervention uptake and sustainability, particularly in digital health contexts [57]. A further rationale for involving end users comes from the potential mismatch between the content commonly addressed in educational interventions and patients’ reported priorities. While the literature frequently focuses on the management of specific complications, such as oral mucositis [58,59] and the prevention of respiratory viral infections [60], topics such as sexuality and palliative care receive comparatively less attention despite being identified by patients as areas of concern [47,61]. These discrepancies further suggest that involving patients and caregivers in intervention development may help align educational content with both clinical priorities and the informational needs, experiences, and values of those directly affected.

A further important finding of this review concerns the limited attention given to caregivers within nursing educational research in the ASCT setting. Although caregiver involvement emerged from the qualitative synthesis as a relevant aspect of the educational process, only one included report directly collected data from caregivers, and no quantitative caregiver-reported outcome or experience data were available. This discrepancy between the recognised importance of caregiver involvement and their limited representation in the available evidence points to a substantial gap in our understanding of their educational experiences and needs in the ASCT setting. Previous research has shown that caregivers of patients undergoing hematopoietic stem cell transplantation may experience substantial caregiving burden, psychological distress, and deterioration in quality of life [62]. Nevertheless, studies specifically examining structured nursing educational interventions targeting caregivers in this setting remain scarce [63]. This gap is particularly relevant since caregivers have their own informational and emotional needs that require specific attention, in addition to being essential in supporting patients’ self-care and treatment adherence [64]. Taken together, these observations reinforce the importance of recognising caregivers not solely as a resource for patients, but as individuals with distinct educational and supportive needs throughout the transplant trajectory. Recent evidence from oncology settings further suggests that caregiver education may contribute to the progressive development of knowledge and relational competencies needed to understand and negotiate the caregiving role [65]. In exploring approaches to address these needs, *Health Information Technology* has been investigated as a potentially flexible and accessible means of delivering caregiver education, allowing asynchronous access to educational resources that may better accommodate caregivers’ schedules and responsibilities [66,67].

While caregivers represent an often-overlooked population, the literature also raises important considerations regarding the characteristics of the transplant populations included in the studies. Notably, many studies report educational interventions conducted on mixed populations of autologous and allogeneic transplant recipients [68,69,70]. However, although these procedures overlap in some aspects of care and complications, they differ in terms of risk profiles, treatment courses, and psychological impacts. Patients undergoing ASCT typically experience a shorter recovery, fewer long-term complications, and no risk of GVHD [71,72,73]. As a result, in this population, nursing educational interventions might focus on managing acute side effects, promoting self-care, and preparing for discharge and the subsequent recovery period. Allogeneic transplant recipients, on the other hand, face a longer and more complex trajectory, marked by greater immunosuppression and the risk of late complications such as chronic GVHD [72,74]. Thus, in this context, educational support might include long-term monitoring, psychological adjustment, and strategies for managing uncertainty over time. These differences suggest that educational needs and experiences may not be fully comparable across transplant populations. Consequently, combining autologous and allogeneic recipients in a single analysis requires careful consideration, as it may obscure meaningful differences in educational priorities, intervention characteristics, and outcomes.

A further issue concerns the visibility of nurses’ contribution to patient education. Although patients frequently report that educational aspects of their care are primarily managed by nurses [75], nursing involvement is often insufficiently described in published educational intervention studies. During the screening process for this review, several potentially relevant reports were excluded because direct nursing involvement could not be clearly established [76,77,78], even though nurses may have contributed to the interventions described. This underreporting may also reflect the fact that TE is often delivered informally and continuously by nurses during routine clinical practice, rather than as part of structured interventions. Given their proximity to patients throughout the care trajectory and their ability to respond to changing informational and supportive needs, nurses nevertheless occupy a central position in the provision of transplant education [75]. More explicit reporting of nurses’ roles and educational activities would therefore help clarify how nursing education is delivered and facilitate its evaluation in future research.

### Strengths and Limitations

This Mixed-Methods Systematic Review has some limitations that should be acknowledged. First, the evidence base was limited, with only seven reports meeting the inclusion criteria and only one report providing usable quantitative outcome data. This substantially limits the strength of the conclusions that can be drawn regarding the effectiveness of nursing educational interventions in the ASCT setting. However, the scarcity of eligible studies also represents an important finding of the review, reflecting the limited availability of research specifically evaluating educational interventions involving nurses in this population. Furthermore, methodological limitations identified among the quantitative reports, including incomplete reporting of missing data and attrition, small sample sizes, and potential confounding associated with observational designs, further constrain the interpretation of the available findings. Importantly, the only study contributing quantitative outcome data also presented inconsistencies in the reporting of self-efficacy scores, which could not be resolved despite contacting the corresponding author. Together with the limited reporting of adjusted effect estimates, these discrepancies further reduce confidence in the quantitative findings and warrant caution when interpreting the reported improvements. An additional limitation concerns the applicability of this study to the target ASCT population. Although its inclusion was considered plausible based on the clinical and educational context described in the primary report, the transplant type could not be definitively established. Similarly, although the intervention was described as nursing-related, the professional qualification of the individual delivering the education could not be independently confirmed. Together, these uncertainties limit the applicability of the quantitative findings to nursing educational interventions in the ASCT setting. Limitations also emerged in the qualitative evidence, which was based on a limited number of independent studies, as two of the three contributing reports originated from the same study. Although all four categories were supported by findings from both independent studies, the limited evidence base should be considered when interpreting the synthesised findings and their transferability to other ASCT settings and populations.

Second, caregivers were scarcely represented in the included literature, with only one report directly collecting data from caregivers and no quantitative data derived from CROMs or CREMs available. This limited the extent to which caregiver-specific experiences and outcomes could be synthesised. At the same time, their limited representation constitutes an evidence gap identified by the review rather than solely a methodological limitation of the review itself. Similarly, the limited use of validated PROMs, CROMs, PREMs, and CREMs across the included literature restricted the quantitative evaluation of educational interventions and the ability to capture outcomes and experiences that are directly relevant to patients and caregivers.

The heterogeneity of study designs, populations, interventions, and outcome assessment, together with the scarcity of usable quantitative data, precluded meta-analysis. Nevertheless, this heterogeneity enabled the review to map a range of educational approaches and intervention characteristics across the available literature. A further strength lies in the use of a convergent segregated mixed-methods approach, which enabled quantitative evidence on intervention outcomes to be considered alongside qualitative evidence on patients’ and caregivers’ experiences. This broader examination of the literature also helped identify recurrent reporting limitations, particularly the inclusion of both autologous and allogeneic transplant recipients and the lack of explicit reporting of transplant type in some studies.

Additional limitations concern potential biases affecting the available evidence. Restricting eligibility to peer-reviewed publications in English or Italian, together with the absence of systematic searches of grey literature and trial registries, may have introduced publication and language biases, potentially resulting in the omission of relevant studies.

Although the statistical assessment of publication bias was prospectively planned, it could not be performed because only one report provided usable quantitative outcome data. Furthermore, selective outcome reporting was not formally assessed. Exploratory searches conducted during the revision process did not identify publicly available protocols or registrations corresponding to the included studies; however, the absence of identifiable protocols does not rule out selective reporting. These limitations should be considered when interpreting the completeness and reliability of the available evidence.

Nevertheless, the review followed a rigorous methodological process in accordance with JBI methodology and PRISMA reporting guidance. Despite the limited evidence base, the integration of quantitative and qualitative findings allowed intervention outcomes to be considered alongside patients’ and caregivers’ experiences, helping to identify relevant educational characteristics and highlight gaps that warrant further investigation.

## 6. Conclusions

This Mixed-Methods Systematic Review highlights the limited evidence currently available on nursing educational interventions for adult oncohaematological patients undergoing ASCT and their caregivers. Quantitative findings remain insufficient to establish intervention effectiveness, while the qualitative synthesis, although based on only two independent studies, offers preliminary insights into educational characteristics valued by patients and caregivers, including personalised information, emotionally responsive delivery, caregiver involvement, and peer support. These findings suggest that moving beyond traditional, predominantly provider-driven educational models towards more flexible and person-centred approaches, responsive to patients’ and caregivers’ lived experiences, evolving needs, and preferences, may represent a promising direction for future intervention development. The review also identified important gaps in the existing evidence base, particularly the underrepresentation of caregivers, the infrequent use of validated PROMs, CROMs, PREMs, and CREMs, and the scarcity of evidence specifically addressing nursing educational interventions in the ASCT setting. Overall, these findings provide a more comprehensive understanding of the current evidence and highlight the importance of considering both informational and experiential dimensions when developing nursing educational interventions across the ASCT trajectory.

### Implications for Practice and Future Research

From a clinical perspective, the findings of this review suggest that nursing education in the ASCT setting should extend beyond the provision of information and consider how educational content is delivered, revisited, and adapted throughout the transplant trajectory. Given their proximity to patients and their continuous involvement in care, nurses are well positioned to support educational processes that respond to changing informational and emotional needs. Flexible and multimodal approaches, combining face-to-face education with printed, digital, or multimedia resources, may offer opportunities to adapt information delivery and facilitate access to educational content over time. Greater consideration should also be given to caregiver involvement and, where appropriate, opportunities for peer support. Furthermore, moving beyond predominantly provider-driven models towards participatory approaches, including co-design [53] and human-centred design [54], may support the development of educational interventions that are more closely aligned with patients’ and caregivers’ priorities, preferences, and lived experiences.

Future research should prioritise rigorous and transparently reported study designs specifically evaluating nursing educational interventions in the ASCT setting. Greater attention should be given to the systematic inclusion of caregivers and to the assessment of caregiver-specific outcomes and experiences. The consistent use of validated PROMs, CROMs, PREMs, and CREMs would strengthen the evaluation of both outcome and experiential dimensions of educational interventions and facilitate comparisons across studies. Future studies should also investigate structured, theory-informed, and participatory educational interventions, including the potential contribution of digital and multimedia modalities. Mixed-methods designs may be particularly valuable in this context, enabling intervention outcomes to be examined alongside patients’ and caregivers’ experiences and perceptions. Overall, strengthening the evidence base will require moving beyond predominantly descriptive and fragmented evidence towards the rigorous development and evaluation of nursing educational interventions that are responsive to the specific needs of patients undergoing ASCT and their caregivers.

## Figures and Tables

**Table 1:** Methodological quality appraisal of the included studies.

Study	Study Design	Screening Questions		Methodological Quality Criteria				
		S1	S2	1	2	3	4	5
**Wang et al., 2023** **[46]**	Quantitative non-randomized	Y	Y	Y	Y	Can’t tell	Can’t tell	Y
**Wu et al., 2025** **[48]**	Quantitative non-randomized	Y	Y	Can’t tell	Y	Can’t tell	N	Can’t tell
**Mank & Molenaar, 2008 [47]**	Quantitative descriptive	Y	Y	Y	Can’t tell	Can’t tell	N	Y
**Salvador, 2006** **[45]**	Quantitative descriptive	Y	Y	Y	Can’t tell	Can’t tell	Y	Y
**Halpin & Konomos, 2022 [42]**	Qualitative	Y	Y	Y	Y	Y	Y	Y
**Halpin, 2026** **[43]**	Qualitative	Y	Y	Y	Y	Y	Y	Y
**Tarzian et al., 1999** **[44]**	Qualitative	Y	Y	Y	Y	Y	Y	Y

Abbreviations: Y = Yes; N = No.

**Table 2:** Characteristics and descriptive summary of all included studies.

Study	Aims	Methods	Participant Details	Characteristics of the Educational Interventions				Results
				Format	Contents	Duration/Frequency	PROMs, CROMs, PREMs, CREMs.	
**Wang et al., 2023** **[46]**	To determine the effect of health promotion strategies on the self-efficacy of patients undergoing bone marrow transplantation in self-care.	**Quantitative Study** Non-randomized clinical trial.	**Patients** *n* = 60 **Intervention Group** *n* = 30 **Comparison Group** *n* = 30	**Intervention Group:** - Individual sessions. - One pre-transplant in-person session, with an educational booklet and researcher’s phone contact for questions. **Comparison Group:** - Educational approach (individual vs. group) not specified. - Routine care and education (not specified).	**Intervention Group:** Health promotion strategies for: - Stress reduction; - Adaptability; - Decision-making; - Enjoyment of life; - Activity; - Rest; - Nutrition; - Medication use. **Comparison Group:** - Not specified.	**Intervention Group:** Not reported. **Comparison Group:** Not reported.	SUPPH Questionnaire.	- A statistically significant increase in compatibility (13.86 ± 3.63 vs. 16.36 ± 2.99, *p* = 0.001), decision-making (10.06 ± 2.72 vs. 11.60 ± 2.06, *p* = 0.001), stress reduction (17.83 ± 5.36 vs. 22.23 ± 3.22, *p* = 0.001), and total self-efficacy (81.36 ± 18.59 vs. 91.23 ± 14.62, *p* = 0.002) in the intervention group. - No significant improvement in life enjoyment in the intervention group (39.60 ± 9.51 vs. 40.96 ± 8.72, *p* = 0.160). - No statistically significant changes in the control group (*p* > 0.05 for all subscales and total self-efficacy score).
**Mank & Molenaar, 2008** **[47]**	To assess the acceptability of an interactive CD-ROM providing information on SCT.	**Quantitative Study** One-group post-test-only design.	**Patients** *n* = 62 82% of enrolled patients (51/62) completed the audit questionnaire	- Individual sessions. - After the standard pre-SCT consultation, patients received an interactive CD-ROM with photos, animations, videos, text, and verbal explanations from a haematologist and fellow patients. Content (printable) was divided into two sections and could be used according to individual preferences.	**First Section:** - Diagnosis and available treatments; - Hospital environment and its organization; - Stem cell transplantation (preparation, procedures, chemo/radiotherapy); - Side effects; - Recovery (infection risk, physical well-being, blood control, and medications); - Going home (monitoring, practical advice, nutrition, patient association, and support). **Second Section:** Videos featuring interviews with former stem cell transplant patients, selected to reflect a diverse range of ages, sexes, and diagnoses, offering a broad overview of experiences.	Questionnaires were to be returned within three weeks of CD-ROM receipt.	Study-specific questionnaire; evidence of validity and reliability was not reported.	Patients rated the CD-ROM as interesting/very interesting (50; 98%), with some finding it encouraging (19; 37%) or comforting (13; 25%), though a minority reported it discouraging (2; 4%) or distressing (11; 22%). The interactive format was judged good/excellent (45; 88%), and most (41; 80%) would recommend it to peers.
**Salvador, 2006** **[45]**	To describe the development of an unit-based oral care guide to better manage or prevent the occurrence and severity of oral mucositis.	**Quantitative Study** Descriptive quality improvement study.	**Patients** *n* = 10	- Individual sessions. - On admission (day 1 of chemotherapy), nurses assessed patients’ knowledge and self-care, reviewed the oral care regimen and its goals with patients and families, and posted guides in all transplant rooms. Patients were monitored daily by staff on adherence to the goals.	**Oral Care Guide**: - Frequency and approach to oral hygiene (brushing, rinsing and flossing): - Recommendations for patients with dentures; - Management of mouthwash; - Medical support for lip and mouth moisturizer, topical anesthetic, or systemic analgesia. **Goals of oral care**: - Maintenance of a clean, moist, and infection-free mouth; - Adherence to the oral care guide; - Adequate hydration, nutrition, and oral comfort.	Not reported.	Not used.	- 6 (60%) patients had either no symptoms of oral mucositis or mild mucositis, continued eating and drinking and did not require pain medication. - 1 (10%) patient discontinued mouthwash due to nausea/vomiting. - 3 (30%) patients developed moderate to severe mucositis, had difficulty eating or drinking, and required systemic analgesia. - Most of the patients tolerated the mouthwash and even exceeded the recommended frequency of rinsing.
**Wu et al., 2025** **[48]**	To evaluate the effectiveness of various oral care regimens during the high-dose conditioning phase of Melfalan followed by ASCT.	**Quantitative Study** Retrospective observational study.	**Patients** *n* = 87 Observation Group *n* = 38 Control Group *n* = 49	- Oral care plans delivered during ASCT conditioning. - During hospitalization, nursing staff supervised adherence and technique through direct observation, real-time feedback, and corrective instruction. Additional support was provided for non-adherent patients through video-based instruction and hands-on coaching.	**Control Group:** Standard oral care: - Chlorhexidine-based oral hygiene; - Mouth rinsing with multiple solutions; - Use of rehabilitation solution; - Cryotherapy. **Observation Group:** Enhanced oral care included standard care plus instruction on a standardized gargling technique (30-s gargling with repeated cycles, cheek puffing, and oropharyngeal involvement).	Continuous nursing supervision during hospitalization, including adherence monitoring and technique support.	Not used.	- The observation group had a significantly shorter hospitalization time than the control group (19.53 ± 0.33 vs. 21.76 ± 0.63, t/χ2 = 2.868, *p* < 0.05). - The weight change in the observation group was significantly less than in the control group (2.51 ± 0.16 vs. 4.24 ± 0.20, t/χ2 = 6.427, *p* < 0.05). - The observation group demonstrated higher nutritional scores (3.32 ± 0.14 vs. 3.04 ± 0.03, t/χ2 = 0.864, *p* < 0.05). - No significant differences were observed in pain scores, mucositis severity, or healing time.
**Halpin & Konomos, 2022** **[42]**	To document the iterative development of a supplemental video-based education program over 18 months for patients with MM undergoing ASCT.	**Qualitative Study** Ethnographic-based iterative formative evaluation plus semi-structured interviews completed several weeks following the education session.	**Ethnographically observed education sessions:** *n* = 70 Semi-structured interviews: **Patients/Caregiver** *n* = 35 *(separate participant counts not reported)* **Nurses/Social Workers** *n* = 7	- Individual sessions. - The R4T was a supplemental video-based education program emailed to patients before their in-person pre-admission education session. At the session’s end, patients received a folder with documents, the treatment schedule, and a book titled “*Autologous Stem Cell Transplantation: A Guide for Patients*”.	**R4T Videos:** - ASCT overview; - Overview of what to expect during and after transplant; - Subcutaneous injections; - Central line maintenance. **In-person education session:** - ASCT overview; - Overview of what to expect during and after transplant; - Discussion and demonstration of self-injection of G-CSF; - Discussion and demonstration of the central venous catheter-related procedures; - Consent for stem cell storage.	**R4T Videos**: 1 h (4 educational videos). **In-person education session**: 90 min.	FACT-G7; data not reported.	The formative evaluation helped identify barriers and facilitators to the patient’s educational experience. **Barriers:** - Fear of what to expect in ASCT; - Fear of what to expect at education visit; - Overwhelming volume of information; - Lack of medical expertise; - Disengaged patients. **Facilitators**: - Large social networks willing to help; - Clinician adjusting script to meet unique needs of patient.
**Halpin, 2026** **[43]**	To examine how cognitive load and socio-emotional adaptation shape individual learning in ASCT.	**Qualitative Study** Interpretative phenomenological analysis.	Same underlying study as Halpin & Konomos [42]; participant details reported above	Same intervention as previously described [42].	Same intervention as previously described [42].	Same intervention as previously described [42].	Same intervention as previously described [42].	Four themes emerged: - Information overload and emotional distress; - Social support and clinical relationships; - Tailored education as a cognitive load reducer; - Perceived control and emotional readiness.
**Tarzian et al., 1999** **[44]**	To explore patients’ perceived information needs during ABMT, before and after the implementation of a clinical pathway including a patient teaching protocol.	**Qualitative Study** Phenomenological inquiry conducted through individual interviews.	**Only patients***n* = 20 Pre-pathway interviews *n* = 10 Post-pathway interviews *n* = 10	**Post-pathway education:** - Educational approach (individual vs. group) not specified. - Patient education was delivered in a structured manner through the implementation of a patient teaching protocol, with the possibility to tailor information to individual patient needs. Education was provided by a multidisciplinary team and included the use of a patient information booklet. **Pre-pathway education**: - Educational approach (individual vs. group) not specified. - Patient education was informal and unstructured.	Not reported.	Not reported.	Not used.	- Pre-pathway patients reported dissatisfaction with insufficient information on procedures and symptoms. - Post-pathway patients reflected on the burden of receiving too much information. - Both groups stressed the importance of family involvement, though some found explaining details to relatives burdensome. - Both highlighted the value of contact with ABMT survivors, though some felt physicians discouraged it due to concerns about side-effect discussions. - Both groups underscored the challenge of balancing information according to patients’ differing needs.

Abbreviations: ABMT = Autologous Bone Marrow Transplantation; ASCT = Autologous Stem Cell Transplantation; FACT-G7 = Functional Assessment of Cancer Therapy—7 items; G-CSF = Granulocyte-Colony Stimulating Factor; R4T = Ready for Transplant; SCT = Stem Cell Transplantation; SUPPH = Strategies Used by People to Promote Health. Note: This table summarizes all included reports for descriptive purposes. Numerical findings are presented regardless of their eligibility for the quantitative outcome synthesis, which required documented evidence of instrument validity or reliability.

**Table 3:** Synthesised findings, categories, and findings from primary reports with related illustrations.

Synthesised Finding	Category	Findings	Credibility	Illustrations	Citation
**Personalisation of content and emotional management of information delivery shape how nursing educational interventions are experienced**	Need for personalised and balanced information	A range of needs for information	U	“I didn’t want to get into too much information for fear I’d … be on overload and it may never happen anyway. I just figure I’m ready for whatever will be will be”. “I’m kind of a question-asking type person. So whenever I wanted to know something, I would ask, ‘What do we do next? What’s going on now?’ I don’t like surprises. I like to know what they expect of me, and I try and comply with that”.	[44]
		Everybody’s different—a fine balance	U	“It’s different personalities, different people, you know. Some people just want to go get it done. They don’t want to be scared. Other people want to know … what’s going on”. “I don’t know how you give information…. We’re all different, I’m sure, and respond differently, and all of us, I think, go into this from a different place: some more debilitated than others, some in better physical shape than others, some younger, some older”.	[44]
		Overwhelming volume of information	U	“They are throwing everything at me at once”.	[42]
		Social support and clinician relationships	U	“The nurse slowed down and made sure I understood. That helped”. “I left that appointment feeling like I hadn’t really learned anything. The doctor was in and out so fast, I barely had time to ask my questions”.	[43]
		Lack of medical expertise	U	“Why do not we stay in the hospital during all of this? This is a lot to put on the patient. We’re not nurses”. Magdalena, a 70-year-old black female, talked about how the videos helped to build her confidence with completing the subcutaneous injections at home, stating “so, she really broke it down and then she showed me them videos and we brought it home, and I said okay, I can do this, it’s not difficult”.	[42]
		Tailored education as a cognitive load reducer	U	“I watched the video before my appointment, so I felt like I already knew what they were talking about. That made a huge difference”. “I went into my appointment not knowing what to expect. It would have helped if I had something to read or watch ahead of time”.	[43]
	Emotional impact of educational interventions	The need to know and the fear of knowing	U	“That’s a scary five pieces of paper…. I’d read enough that I was aware that the things they were mentioning weren’t a necessity, that you were going to have a liver shut down, but if somebody isn’t already informed, to take home the informed consent, if you’re already scared, would terrify me”. “Some of the words that were used were ‘discomfort’, ‘you won’t have an appetite’ when in fact it isn’t ‘you won’t have an appetite’; it’s ‘you will be throwing up constantly’. It isn’t ‘discomfort’; it’s ‘pain’”.	[44]
		Anxiety surrounding what to expect in ASCT	U	During her interview in the hospital she described constant concern about what might lie ahead in her treatment journey and, “what might be around the corner”. Emma expressed surprise that she could receive her stem cells just 2 days after receiving a high dose of chemotherapy (i.e., melphalan). She was worried that the chemotherapy would kill the stem cells—and did not understand that the previous 24 h was enough for the chemotherapy to pass through her system.	[42]
		Perceived control and emotional readiness	U	“The doctor gave me options and let me ask a lot of questions. Because I had a say, I actually remembered more of what they told me”. “They already decided for me. I felt like I was just being told what to do, and none of it stuck”.	[43]
**Caregiver involvement and peer support are perceived as important components of the educational process**	Peer interaction can reduce anxiety and uncertainty	Someone who has been there	**U**	“I didn’t need to know that. I needed to talk with someone who’d been through it”. “Don’t be afraid to let patients talk to other patients. It’s what we need”. She said, however, that she would be selective in choosing a recovered ABMT patient, one who would be “accurate and yet fair”. “Because I was too scared to say anything at that point. What I did was run home and call somebody who had already been there”.	[44]
		Anxiety surrounding what to expect at the education visit	**C**	She also has been engaged in online groups (Facebook, Smart Patients Team, and Myeloma Group) and she finds those to be useful for hearing about what others have experienced. She did not feel comfortable posting on these herself and was concerned about the reliability of what she read, especially concerning that people with bad experiences or opinions of ASCT were more likely to post, still she found them helpful.	[42]
	Active involvement of caregivers in educational interventions	The burden of teaching family	**U**	“I wish a little bit more communication with my family, my sister, and my husband, and even my daughter at her own level could have been made so that when I did come home, they would realize that, especially after three weeks, that I was basically still in the hospital”.	[44]
		Large social networks willing to help	**C**	Scott, a 69-year-old white male, stated during an ethnographic interview in his hospital room that he appreciated the videos and that he and his family “watched them multiple times”. One caregiver, the daughter of a 70 year-old black female, stated, “And I just had to like educate myself by the time the new written materials came up. I was like I just cannot read all of this. You know it is just so much information at one time that you have to keep going but you have to have it—so I just tried to learn the basics and the basics to me was the port, was the line”.	[42]
		Social support and clinician relationships	**U**	“My daughter asked questions I wouldn’t have thought of”. “My wife took notes so I could focus on listening. Later, we talked through everything together”.	[43]
		Disengaged patients	**Not** **Supported**	-	[42]

Abbreviations: C = Credible; U = Unequivocal.

## Data Availability

No new data were created or analyzed in this study. Data sharing is not applicable to this article.

## Supplementary Materials

The following supporting information can be downloaded at: https://www.mdpi.com/article/10.3390/healthcare14213693/s1, Supplementary Table S1: Search strategy; Supplementary Table S2: MMAT appraisal criteria and supporting explanations; Supplementary File S1: Reports excluded after full-text review with reasons. References [79,80,81,82,83,84,85,86,87,88,89,90,91,92] are cited in Supplementary File S1.

## Abbreviations

The following abbreviations are used in this manuscript:

ASCT	Autologous Stem Cell Transplantation
CREMs	Caregiver-Reported Experience Measures
CROMs	Caregiver-Reported Outcome Measures
FACT-G7	Functional Assessment of Cancer Therapy—7 items
GVHD	Graft-versus-Host Disease
MMAT	Mixed Methods Appraisal Tool
PEO	Population, Exposure, Outcome
PICO	Population, Intervention, Comparison, Outcome
POEMS	Polyneuropathy–Organomegaly–Endocrinopathy–Monoclonal gammopathy–Skin changes
PREMs	Patient-Reported Experience Measures
PRISMA	Preferred Reporting Items for Systematic Reviews and Meta-Analyses
PROMs	Patient-Reported Outcome Measures
RCTs	Randomized Controlled Trials
SUPPH	Strategies Used by People to Promote Health
TE	Therapeutic Education
