# Supplementary material for: Nursing Educational Interventions for Oncohaematological Patients Undergoing Autologous Stem Cell Transplantation and Their Caregivers: A Mixed-Methods Systematic Review
Source: Healthcare (Basel). 2026 Nov 1;14(21):3693. doi: 10.3390/healthcare14213693 (PMC13763544; doi:10.3390/healthcare14213693)
Supplement: Supplementary file 1 [file healthcare-14-03693-s001.zip › Supplementary File S1. Reports excluded after full-text review with reasons.pdf]

Supplementary File S1. Reports excluded after full-text review with reasons

Identification of studies via databases and registers

Reason for exclusion: Report not retrieved (n= 2).

1. Cromer JM. Care, support and education. *On Call*. 2005;8(5):22–5. [79]
2. Walker FE, Roethke SK, Sandman V, Clark K, Martin G. Guiding patients and their families through peripheral stem cell transplantation with the help of a teaching booklet. Oncol Nurs Forum. 1994 Apr;21(3):585-91. PMID: 8052554. [80]

Reason for exclusion: Wrong Study Design (n= 2).

1. **[Protocol]** Raje NS, Nadeem O, Mikhael J, Ludwig C, Agarwal A, Inumerable J, Ong TJ, Jagannath S. Pilot Trial of the My Hematology Oncology Patient Experience (MyHOPE™) for Multiple Myeloma digital solution in patients with MM. *Blood*. 2020;136(Suppl 1):3. doi:10.1182/blood-2020-141591. [81]
2. **[Abstract]** Jarden M, Holder J, Hovgaard D, Adamsen L. The active patient (TAP): a qualitative study of the patients' experience and appraisal of a multimodal rehabilitation intervention during hematopoietic stem cell transplantation. Psycho-Oncology. 2014;23:129–130. [82]

Reason for exclusion: Wrong Language (n= 1).

1. **[Chinese Language]** Chen Y, Wang H. Psychological reactions of patients underwent hematopoietic stem cell transplantation and nursing research. Chinese Nursing Research. 2004;18(7B):1230–1. [83]

Reason for exclusion: Wrong Population (n= 10).

1. **[Autologous and allogeneic mixed transplant population]** Fauer AJ, Hoodin F, Lalonde L, Errickson J, Runaas L, Churay T, Seyedsalehi S, Warfield C, Chappell G, Brookshire K, Chaar D, Shin JY, Byrd M, Magenau J, Hanauer DA, Choi SW. Impact of a health information technology tool addressing information needs of caregivers of adult and pediatric hematopoietic stem cell transplantation patients. Support Care Cancer. 2019 Jun;27(6):2103-2112. doi: 10.1007/s00520-018-4450-4. Epub 2018 Sep 20. PMID: 30232587; PMCID: PMC6431273. [66]
2. **[Autologous and allogeneic mixed transplant population]** Sarıtürk Ç, Gereklioğlu Ç, Korur A, Asma S, Yeral M, Solmaz S, Büyükkurt N, Tepebaşı S, Kozanoğlu İ, Boğa C, Özdoğu H. Effectiveness of Visual Methods in Information Procedures for Stem Cell Recipients and Donors. Turk J Haematol. 2017 Dec 1;34(4):321-327. doi: 10.4274/tjh.2016.0118. Epub 2016 Jul 15. PMID: 27476890; PMCID: PMC5774375. [51]
3. **[Autologous and allogeneic mixed transplant population]** Yamagata K, Arai C, Sasaki H, Takeuchi Y, Onizawa K, Yanagawa T, Ishibashi N, Karube R, Shinozuka K, Hasegawa Y, Chiba S, Bukawa H. The effect of oral management on the severity of oral mucositis during hematopoietic SCT. Bone Marrow Transplant. 2012 May;47(5):725-30. doi: 10.1038/bmt.2011.171. Epub 2011 Aug 29. PMID: 21874059. [59]
4. **[Autologous and allogeneic mixed transplant population]** Kemp J, Dickerson J. Interdisciplinary modular teaching for patients undergoing progenitor cell transplantation. Clin J Oncol Nurs. 2002 May-Jun;6(3):157-60. doi: 10.1188/02.CJON.157-160. PMID: 11998609. [68]
5. **[Autologous and allogeneic mixed transplant population]** McErlean G, Ashley C, Pradhan A, Paterson A, Farnham G, Owen F, et al. "It's the Ride of Your Life": A Qualitative Study on Survivor Awareness and Understanding of Blood and Marrow Transplantation. Seminars in Oncology Nursing. 2025 Feb;41(1):151756. doi:10.1016/j.soncn.2024.151756. [75]
6. **[Allogeneic transplant population]** Metoyer LJ. Education of hematopoietic stem cell transplant caregivers in preparation for their role. J Adv Pract Oncol. 2013 Nov;4(6):432-7. doi: 10.6004/jadpro.2013.4.6.6. PMID: 25032023; PMCID: PMC4093455. [31]

7. **[Hematologic and non-hematologic, malignant and non-malignant mixed population]** Ferguson PE, Jordens CF, Gilroy NM. Patient and family education in HSCT: improving awareness of respiratory virus infection and influenza vaccination. A descriptive study and brief intervention. Bone Marrow Transplant. 2010 Apr;45(4):656-61. doi: 10.1038/bmt.2009.209. Epub 2009 Aug 17. PMID: 19684629. [60]
8. **[Mainly ≤18 years old (*inferred*)]** Jalal R, Shahrzad G, Roya A, Masoumeh Z. The effect of self-care education with smart phone applications on the severity of nausea and vomiting after stem cell transplantation in leukemia patients. Hematol Transfus Cell Ther. dicembre 2024;46 Suppl 6(Suppl 6):S144–9. [84]
9. **[Autologous and allogeneic mixed transplant population]** Gray TF, Revette AC, Nava-Coulter B, Gould K, Close S, Sullivan LM, Flynn KE, Katsetos KA, Cutler C, Ho VT, Hammer MJ, El-Jawahri A, Tulsky JA. Caregiver experiences managing information prior to hematopoietic stem cell transplantation and after transition to home: a qualitative study. Support Care Cancer. 2025 Apr 22;33(5):402. doi: 10.1007/s00520-025-09445-2. PMID: 40258969. [25]
10. **[Autologous and allogeneic mixed transplant population]** Applebaum AJ, Gebert RR, Manschot C, Kuhn E, Laber E, Kastrinos A, Ansah OO, Mitchell HR, Smith SK. A Pilot Study of the Cancer Distress Coach-Caregiver App: A Digital Intervention for Reducing PTSD Symptoms in HCT Caregivers. Psychooncology. 2025 Oct;34(10):e70305. doi: 10.1002/pon.70305. PMID: 41116260; PMCID: PMC12880602. [85]

Reason for exclusion: **Wrong Intervention (n= 9).**

1. **[No nursing involvement in intervention delivery]** Macleod F, Pink J, Beattie S, Feldstain A. Program Evaluation of a Class Addressing Psychosocial Topics in Preparation for Hematopoietic Cell Transplantation: a Brief Report. J Cancer Educ. 2023 Feb;38(1):60-65. doi: 10.1007/s13187-021-02078-z. Epub 2021 Aug 13. PMID: 34387833. [69]
2. **[Post-ASCT intervention]** Mohamed AF, Soliman N, Abd-El-Moneem El-Sayied H, Talaat A. Nursing Intervention for Caregivers of Post Autologous Bone Marrow Transplantation Patients at Home. Medico-Legal Update. 2021;21(1):112–7. [86]
3. **[Post-ASCT intervention]** Kazempour M, Keshtkaran Z, Vizeshfar F. The effect of training on self-efficacy of patients with leukemia after hematopoietic stem cell transplantation. Archivos Venezolanos de Farmacologia y Terapeutica. 2021;40(2):136–40. [52]
4. **[Post-ASCT intervention]** Heermann JA, Eilers JG, Carney PA. Use of modified OSCEs to verify technical skill performance and competency of lay caregivers. J Cancer Educ. Summer 2001;16(2):93–8. [87]
5. **[Psychological intervention]** Amonoo HL, Newcomb R, Lorenz KA, Psenka R, Holmbeck K, Farnam EJ, Tse A, Desai S, Vassev N, Waldman LP, El-Jawahri A. A novel psychosocial virtual reality intervention (BMT-VR) for patients undergoing hematopoietic stem cell transplantation: Pilot randomized clinical trial design and methods. Contemp Clin Trials. 2024 Jul;142:107550. doi: 10.1016/j.cct.2024.107550. Epub 2024 Apr 27. PMID: 38685401; PMCID: PMC11180582. [88]
6. **[Psychological intervention]** Pensak NA, Joshi T, Simoneau T, Kilbourn K, Carr A, Kutner J, Laudenslager ML. Development of a Web-Based Intervention for Addressing Distress in Caregivers of Patients Receiving Stem Cell Transplants: Formative Evaluation With Qualitative Interviews and Focus Groups. JMIR Res Protoc. 2017 Jun 22;6(6):e120. doi: 10.2196/resprot.7075. Erratum in: JMIR Res Protoc. 2017 Aug 15;6(8):e130. doi: 10.2196/resprot.8322. PMID: 28642213; PMCID: PMC5500777. [67]
7. **[Cryotherapy-focused intervention]** Salvador P, Azusano C, Wang L, Howell D. A pilot randomized controlled trial of an oral care intervention to reduce mucositis severity in stem cell transplant patients. J Pain Symptom Manage. 2012 Jul;44(1):64-73. doi: 10.1016/j.jpainsymman.2011.08.012. Epub 2012 Jun 5. PMID: 22672917. [58]

8. **[Non-educational intervention]** Halpin SN, Konomos M, Jowers I. Interrupted Identities: Autologous Stem Cell Transplant in Patients With Multiple Myeloma. J Patient Exp. 2021;8:2374373521998864. [89]
9. **[No nursing involvement in intervention delivery]** Henley EC, Liphart HA, Morris KJ, Awoyinka I, Irwin MR, Costanzo ES, Winston D, D'Souza A, Stolley M, Dhakal B, Mohan M, Pasquini MC, Cole SW, Doerwald ES, Bendis PC, Rentscher KE, Rumble ME, Szabo A, Rao S, Knight JM. Mindfulness vs. sleep education during autologous hematopoietic cell transplantation for multiple myeloma: Feasibility of a randomized controlled pilot study. Contemp Clin Trials Commun. 2025 Aug 18;47:101540. doi: 10.1016/j.conctc.2025.101540. PMID: 40893725; PMCID: PMC12397865. [90]

Reason for exclusion: Insufficient additional information (n= 1).

1. Halpin SN, Konomos M. Implementing Video-Based Education for Multiple Myeloma Patients Preparing for Autologous Stem Cell Transplant: A RE-AIM Evaluation. J Cancer Educ. 2025 Aug 10. doi: 10.1007/s13187-025-02692-1. Epub ahead of print. PMID: 40783884. [91]

Identification of studies via other methods

Reason for exclusion: **Wrong Intervention (n= 2).**

1. **[Post-ASCT intervention]** Braamse AMJ, van Meijel B, Visser OJ, Boenink AD, Cuijpers P, Eeltink CE, Hoogendoorn AW, van Marwijk Kooy M, van Oppen P, Huijgens PC, Beekman ATF, Dekker J. A randomized clinical trial on the effectiveness of an intervention to treat psychological distress and improve quality of life after autologous stem cell transplantation. Ann Hematol. 2016 Jan;95(1):105-114. doi: 10.1007/s00277-015-2509-6. Epub 2015 Sep 30. PMID: 26420062; PMCID: PMC4700101. [92]
2. **[Post-ASCT intervention]** Wendt C. Perception and Assessment of Verbal and Written Information on Sex and Relationships after Hematopoietic Stem Cell Transplantation. J Cancer Educ. 2017 Dec;32(4):681-689. doi: 10.1007/s13187-016-1054-x. PMID: 27278129. [61]
