# Supplementary material for: Nursing Educational Interventions for Oncohaematological Patients Undergoing Autologous Stem Cell Transplantation and Their Caregivers: A Mixed-Methods Systematic Review
Source: Healthcare (Basel). 2026 Nov 1;14(21):3693. doi: 10.3390/healthcare14213693 (PMC13763544; doi:10.3390/healthcare14213693)
Supplement: Supplementary file 1 [file healthcare-14-03693-s001.zip › Supplementary Table S1. Search Strategy.pdf]

Supplementary Table S1. Search Strategy

Search conducted up to 6 April 2026.

Pubmed

Search syntax	Citations found
("Hematologic Neoplasms"[Mesh] OR "Bone Marrow Neoplasms"[Mesh] OR "Leukemia"[Mesh] OR "Multiple Myeloma"[Mesh] OR "Lymphoma"[Mesh] OR "Hodgkin Disease"[Mesh] OR "Myelodysplastic Syndromes"[Mesh] OR "Lymphoma, Non-Hodgkin"[Mesh] OR "Caregivers"[Mesh] OR "Hemato* Neoplasm"[Title/Abstract] OR "Hemato* Malignancy"[Title/Abstract] OR "Hemato* Cancer"[Title/Abstract] OR "Hemato* Tumor"[Title/Abstract] OR "Haemato* Neoplasm"[Title/Abstract] OR "Haemato* Malignancy"[Title/Abstract] OR "Haemato* Cancer"[Title/Abstract] OR "Haemato* Tumor"[Title/Abstract] OR "Haemato* Tumour"[Title/Abstract] OR "Hemato* Tumour"[Title/Abstract] OR "Blood Cancer*"[Title/Abstract] OR "Bone Marrow Neoplasm*"[Title/Abstract] OR "Bone Marrow Cancer*"[Title/Abstract] OR "Bone Marrow Malignanc*"[Title/Abstract] OR "Bone Marrow Tumor*"[Title/Abstract] OR "Leukemia*"[Title/Abstract] OR "Acute Leukemia*"[Title/Abstract] OR "Chronic Leukemia*"[Title/Abstract] OR "Chronic Myeloid Leukemia*"[Title/Abstract] OR "Acute Myeloid Leukemia*"[Title/Abstract] OR "Acute Lymphocytic Leukaemia*"[Title/Abstract] OR "Chronic Lymphocytic Leukaemia*"[Title/Abstract] OR "Multiple Myeloma*"[Title/Abstract] OR "Multiple-Myeloma*"[Title/Abstract] OR "Myeloma*"[Title/Abstract] OR "Myeloma-Multiple*"[Title/Abstract] OR "Myeloma Multiple*"[Title/Abstract] OR "Hodgkin Disease*"[Title/Abstract] OR "Hodgkin's Disease*"[Title/Abstract] OR "Hodgkins Lymphoma*"[Title/Abstract] OR "Adult Hodgkin Lymphoma*"[Title/Abstract] OR "Lymphoma*"[Title/Abstract] OR "Hodgkin Lymphoma*"[Title/Abstract] OR "Hodgkin's Lymphoma*"[Title/Abstract] OR "Non-Hodgkin's Lymphoma*"[Title/Abstract] OR "Non Hodgkin's Lymphoma*"[Title/Abstract] OR "Non Hodgkin Lymphoma*"[Title/Abstract] OR "Oncohematolog* patient"[Title/Abstract] OR "Oncohaematolog* patient"[Title/Abstract] OR "Caregiver*"[Title/Abstract] OR "Carer*"[Title/Abstract] OR "Family Caregiver*"[Title/Abstract] OR "Famil*"[Title/Abstract] OR "Spouse"[Title/Abstract] OR "Spouse Caregiver*"[Title/Abstract] OR "Informal Caregiver*"[Title/Abstract] OR "Care-giver*"[Title/Abstract] OR "Care Giver*"[Title/Abstract] OR "Parent*"[Title/Abstract] OR "Daughter*"[Title/Abstract] OR "Brother*"[Title/Abstract] OR "Sister*"[Title/Abstract] OR "Sibling*"[Title/Abstract] OR "Offspring*"[Title/Abstract] OR "Kinship"[Title/Abstract] OR "Caregiving"[Title/Abstract])<br>**AND**<br>("Stem Cell Transplantation"[Mesh] OR "Peripheral Blood Stem Cell Transplantation"[Mesh] OR "Hematopoietic Stem Cell Transplantation"[Mesh] OR "ASCT"[Title/Abstract] OR "HSCT"[Title/Abstract] OR "Stem Cell Transplant*"[Title/Abstract] OR "Peripheral Blood Stem Cell Transplant*"[Title/Abstract] OR "Peripheral Stem Cell Transplan*"[Title/Abstract] OR "Autologous hematopoietic stem-cell transplant*"[Title/Abstract] OR "Autologous haematopoietic stem-cell transplant*"[Title/Abstract] OR "Autologous Transplant*"[Title/Abstract] OR "Autologous Stem-Cell Transplant*"[Title/Abstract] OR "Haematopoietic Stem Cell Transplant*"[Title/Abstract] OR "Hematopoietic Stem Cell Transplant*"[Title/Abstract] OR "Autotransplant*"[Title/Abstract] OR "Autotrasplant*"[Title/Abstract])<br>**AND**<br>("Education"[Mesh] OR "Patient Education as Topic"[Mesh] OR "Education"[Title/Abstract] OR "Therapeutic Educat*"[Title/Abstract] OR "Nursing Educat*"[Title/Abstract] OR "Teaching"[Title/Abstract] OR "Training"[Title/Abstract] OR "Learning"[Title/Abstract] OR "Instruction"[Title/Abstract] OR "Counsel*"[Title/Abstract] OR "Patient Educat*"[Title/Abstract] OR "Patient Informat*"[Title/Abstract] OR "Patient Literacy"[Title/Abstract] OR "Patient Training"[Title/Abstract] OR "Patient Counsel*"[Title/Abstract] OR "Patient Instruction*"[Title/Abstract] OR "Consumer Educat*"[Title/Abstract] OR "Consumer Informat*"[Title/Abstract] OR "Consumer Literacy"[Title/Abstract] OR "Consumer Training"[Title/Abstract] OR "Consumer Counsel*"[Title/Abstract] OR "Health Education"[Title/Abstract] OR "Health Information"[Title/Abstract] OR "Health Literacy"[Title/Abstract] OR "Health Program*"[Title/Abstract] OR "Educati* Program"[Title/Abstract] OR "Educati* Intervention"[Title/Abstract] OR "Educati* Training"[Title/Abstract] OR "Information Service"[Title/Abstract] OR "Information Dissemination"[Title/Abstract] OR "Consumer Health Information"[Title/Abstract] OR "Patient Advice"[Title/Abstract] OR "Consumer Advice"[Title/Abstract] OR "Caregiver Informat*"[Title/Abstract]	1139

OR "Caregiver Counsel*"[Title/Abstract] OR "Caregiver Educat*"[Title/Abstract] OR "Caregiver Instruction*"[Title/Abstract] OR "Caregiver Training*"[Title/Abstract] OR "Caregiver Advice"[Title/Abstract])	

Scopus

Search syntax	Citations found
**1** TITLE-ABS-KEY ("Oncoh?matolog* patient")<br>**2** TITLE-ABS-KEY (Care?giver* OR Carer* OR "Family Caregiver*" OR Famil* OR Spouse OR "Informal Caregiver*" OR Parent* OR Daughter* OR Brother* OR Sister* OR Sibling* OR Offspring* OR Kinship OR Caregiving)<br>**3** TITLE-ABS-KEY (("H?emato*") W/1 (Neoplasm* OR Malignanc* OR Cancer* OR Tum?r*))<br>**4** TITLE-ABS-KEY (("Bone Marrow") W/1 (Neoplasm* OR Cancer* OR Malignanc* OR Tum?r*))<br>**5** TITLE-ABS-KEY ("Leukemia*" OR "Multiple Myeloma*" OR Lymphoma* OR "Hodgkin Disease*" OR "Myelodysplastic Syndrome*" OR "Blood Cancer*" OR "Acute Leukemia*" OR "Chronic Leukemia*" OR "Chronic Myeloid Leukemia*" OR "Acute Myeloid Leukemia*" OR "Acute Lymphocytic Leukaemia*" OR "Chronic Lymphocytic Leukaemia*" OR Multiple-Myeloma* OR Myeloma* OR Myeloma-Multiple* OR "Myeloma Multiple*" OR "Hodgkins Disease*" OR "Hodgkins Lymphoma*" OR "Adult Hodgkin Lymphoma*" OR "Hodgkin Lymphoma*" OR "Hodgkins Lymphoma*" OR "Non-Hodgkins Lymphoma*" OR "Non Hodgkins Lymphoma*" OR "Non Hodgkin Lymphoma*")<br>**6** TITLE-ABS-KEY ((burkitt) W/1 (lymphom* OR neoplasm* OR cancer* OR tum?r*))<br>**7** #1 OR #2 OR #3 OR #4 OR #5 OR #6<br>**8** TITLE-ABS-KEY (ASCT OR HSCT OR "Stem Cell Transplant*" OR "Peripheral Blood Stem Cell Transplant*" OR "Peripheral Stem Cell Transplan*" OR "Autologous hematopoietic stem-cell transplant*" OR "Autologous Transplant*" OR "Autologous Stem-Cell Transplant*" OR "Hematopoietic Stem Cell Transplant*" OR Autotransplant*)<br>**9** TITLE-ABS-KEY ((autolog*) W/4 (transplant* OR rescue*))<br>**10** #8 OR #9<br>**11** TITLE-ABS-KEY (Educat* OR Teaching OR Training OR Learning OR Instruction* OR Counsel*ing)<br>**12** TITLE-ABS-KEY ((Therapeutic OR Nursing OR Program* OR Intervention* OR Training*) W/1 (Educat*))<br>**13** TITLE-ABS-KEY ((Teaching OR Training OR Learning) W/1 (Material*))<br>**14** TITLE-ABS-KEY ((Health) W/1 (Education OR Information OR Literacy OR Program*))<br>**15** TITLE-ABS-KEY ((Patient* OR Caregiver* OR Consumer*) W/1 (Educat* OR Informat* OR Literacy OR Training* OR Counsel* OR Instruction* OR Advice*))<br>**16** TITLE-ABS-KEY (("Informati* Service" OR "Informati* Dissemination" OR "Consumer Health Information"))<br>**17** #11 OR #12 OR #13 OR #14 OR #15 OR #16<br>**18 #7 AND #10 AND #17**	2918

CINAHL Complete (EBSCOhost)

Search syntax	Citations found
(MH "Hematologic Neoplasms+" OR MH "Leukemia+" OR MH "Leukemia, Myeloid+" OR MH "Leukemia, Lymphocytic+" OR MH "Leukemia, Myeloid, Acute+" OR MH "Leukemia, Lymphocytic, Acute" OR MH "Leukemia, Lymphocytic, Chronic+" OR MH "Leukemia, Promyelocytic, Acute" OR MH "Leukemia, Hairy Cell" OR MH "Leukemia, Radiation-Induced" OR MH "Leukemia, Erythroblastic, Acute" OR MH "Multiple Myeloma+" OR MH "Bone Marrow Neoplasms" OR MH "Myelodysplastic Syndromes" OR MH "Hodgkin's Disease" OR MH "Lymphoma, Extranodal NK-T-Cell" OR MH "Lymphoma, T-Cell, Cutaneous+" OR MH "Lymphoma, Large-Cell, Anaplastic" OR MH "Lymphoma, B-Cell+" OR MH "Lymphoma, T-Cell+" OR MH "Lymphoma, Non-Hodgkin's+" OR MH "Lymphoma+" OR "Oncohematology patient*" OR "Oncohematological patient*" OR "Hematologic Neoplasm*" OR "Hematologic Malignanc*" OR "Hematologic Cancer*" OR "Hematologic Tumor*" OR "Haematologic Neoplasm*" OR "Haematologic Malignanc*" OR "Haematologic Cancer*" OR "Haematologic Tumor*" OR "Blood Cancer*" OR "Hematopoietic Malignanc*" OR "Hematopoietic Neoplasm*" OR "Hematopoietic Cancer*" OR "Hematopoietic Tumor*" OR "Hematological Neoplasm*" OR "Hematological Malignanc*" OR "Hematological Cancer*" OR "Hematological Tumor*")<br>**AND**<br>(MH "Hematopoietic Stem Cells" OR MH "Hematopoietic Stem Cell Transplantation" OR MH "Bone Marrow Transplantation, Autologous" OR "ASCT" OR "HSCT" OR "Stem Cell Transplant*" OR "Peripheral Blood Stem Cell Transplant*" OR "Stem Cell Transplantation, Peripheral" OR "Transplantation, Peripheral Stem Cell" OR "Peripheral Stem Cell Transplan*" OR "Autologous hematopoietic stem-cell	388

transplant*" OR "Autologous haematopoietic stem-cell transplant*" OR "Autologous Transplantation" OR "Autologous Stem-Cell Transplant*" OR "Autologous Stem Cell Transplant*" OR "Haematopoietic Stem Cell Transplant*" OR "Hematopoietic Stem Cell Transplant*")<br>**AND**<br>(MH "Consumer Health Information+" OR MH "Digital Technology+" OR MH "Digital Health+" OR MH "Digital Media" OR MH "Educational Technology" OR MH "Online Education" OR "Digital Education program*" OR "Digital Technolog*" OR "Online Intervention*" OR "Online Education*" OR "Online Program*" OR "Online Learning" OR "Digital Health" OR "Digital Life Coaching" OR "eHealth" OR "e-Health" OR "E-coach" OR "E-tool" OR "Web-based Intervention*" OR "Web-based Program*" OR "Web-based Education*" OR "Web-based Application*" OR "TeleHealth" OR "Tele-Health" OR MH "Internet-Based Intervention" OR MH "Education+" OR MH "Adult Education" OR MH "Patient Education+" OR MH "Health Education+" OR "Educat*" OR "Teaching" OR "Training" OR "Learning" OR "Instruction*" OR "Counsel?ing" OR (("Therapeutic" OR "Nursing" OR "Program*" OR "Intervention*" OR "Training*") W/1 ("Educat*")) OR (("Teaching" OR "Training" OR "Learning") W/1 ("Material*")) OR (("Health") W/1 ("Education" OR "Information" OR "Literacy" OR "Program*")) OR (("Patient*" OR "Caregiver*" OR "Consumer*") W/1 ("Educat*" OR "Informat*" OR "Literacy" OR "Training*" OR "Counsel*" OR "Instruction*" OR "Advice*")) OR "Informati* Service*" OR "Informati* Dissemination" OR "Consumer Health Information")	
**Search settings:** The search was conducted in CINAHL Complete via EBSCOhost, with the "All Fields" option selected for each search block.	

APA PsycINFO (EBSCOhost)

Search syntax	Citations found
XB ((DE "Blood and Lymphatic Disorders" OR DE "Leukemias" OR DE "Caregivers" OR DE "Caregiving" OR "Oncoh?ematolog* patient*" OR "Caregiver*" OR "Carer*" OR "Family Caregiver*" OR "Famil*" OR "Spouse" OR "Spouse Caregiver*" OR "Informal Caregiver*" OR "Care-giver*" OR "Care Giver*" OR "Parent*" OR "Daughter*" OR "Brother*" OR "Sister*" OR "Sibling*" OR "Offspring*" OR "Kinship" OR "Caregiving" OR (("H?emato*") W/1 ("Neoplasm*" OR "Malignanc*" OR "Cancer*" OR "Tumo*r*")) OR (("Bone Marrow") W/1 ("Neoplasm*" OR "Cancer*" OR "Malignanc*" OR "Tumo*r*")) OR (("burkitt") W/1 ("lymphom*" OR "neoplasm*" or "cancer*" or "tumor*")) OR ("Leukemia*" OR "Multiple Myeloma*" OR "Lymphoma*" OR "Hodgkin Disease*" OR "Myelodysplastic Syndrome*" OR "Blood Cancer*" OR "Acute Leukemia*" OR "Chronic Leukemia*" OR "Chronic Myeloid Leukemia*" OR "Acute Myeloid Leukemia*" OR "Acute Lymphocytic Leukaemia*" OR "Chronic Lymphocytic Leukaemia*" OR "Multiple-Myeloma*" OR "Myeloma*" OR "Myeloma-Multiple*" OR "Myeloma Multiple*" OR "Hodgkin's Disease*" OR "Hodgkins Lymphoma*" OR "Adult Hodgkin Lymphoma*" OR "Hodgkin Lymphoma*" OR "Hodgkin's Lymphoma*" OR "Non-Hodgkin's Lymphoma*" OR "Non Hodgkin's Lymphoma*" OR "Non Hodgkin Lymphoma*))<br>**AND**<br>XB ((DE "Organ Transplantation" OR "ASCT" OR "HSCT" OR "Stem Cell Transplant*" OR "Peripheral Blood Stem Cell Transplant*" OR "Peripheral Stem Cell Transplan*" OR "Autologous hematopoietic stem-cell transplant*" OR "Autologous Transplant*" OR "Autologous Stem-Cell Transplant*" OR "Hematopoietic Stem Cell Transplant*" OR "Autotransplant*" OR ((autolog*) W/4 (transplant* OR rescue*))))<br>**AND**<br>XB ((DE "Education" OR DE "Adult Education" OR DE "Client Education" OR DE "Drug Education" OR DE "Rehabilitation Education" OR DE "Consumer Education" OR DE "Career Education" OR DE "Health Education" OR DE "Electronic Learning" OR DE "Asynchronous Learning" OR DE "Electronic Courses" OR DE "Learning Management Systems" OR DE "Mobile Learning" OR DE "Computer Assisted Instruction" OR DE "Computer Assisted Language Learning" OR DE "Computer Supported Collaborative Learning" OR DE "Digital Game-Based Learning" OR DE "Intelligent Tutoring Systems" OR DE "Health Promotion" OR DE "Disease Management" OR DE "Parent Training" OR "Educat*" OR "Teaching" OR "Training" OR "Learning" OR "Instruction*" OR "Counsel?ing" OR (("Therapeutic" OR "Nursing" OR "Program*" OR "Intervention*" OR "Training*") W/1 ("Educat*")) OR (("Teaching" OR "Training" OR "Learning") W/1 ("Material*")) OR (("Health") W/1 ("Education" OR "Information" OR "Literacy" OR "Program*")) OR (("Patient*" OR "Caregiver*" OR "Consumer*") W/1 ("Educat*" OR "Informat*" OR "Literacy" OR "Training*" OR "Counsel*" OR "Instruction*" OR "Advice*")) OR "Informati* Service*" OR "Informati* Dissemination" OR "Consumer Health Information"))	210

Cochrane CENTRAL Register of Controlled Trials

Search syntax	Citations found
#1 MeSH descriptor: [Caregivers] explode all trees<br>#2 MeSH descriptor: [Parenting] explode all trees<br>#3 MeSH descriptor: [Family Relations] explode all trees<br>#4 caregiver* or carer* or (family NEXT caregiver*) or famil* or spouse or (spouse NEXT caregiver*) or (informal NEXT caregiver*) or caregiver* or parent* or daughter* or brother* or sister* or sibling* or offspring* or kinship or caregiving<br>#5 MeSH descriptor: [Hematologic Neoplasms] explode all trees<br>#6 MeSH descriptor: [Bone Marrow Neoplasms] explode all trees<br>#7 MeSH descriptor: [Leukemia] explode all trees<br>#8 MeSH descriptor: [Multiple Myeloma] explode all trees<br>#9 MeSH descriptor: [Hodgkin Disease] explode all trees<br>#10 MeSH descriptor: [Lymphoma, Non-Hodgkin] explode all trees<br>#11 MeSH descriptor: [Myelodysplastic Syndromes] explode all trees<br>#12 oncoh?ematologic* patient<br>#13 (h?emato* or blood or bone marrow) NEAR/1 (malign* or neoplasm* or cancer* or tumor* or tumour*)<br>#14 leuk?mia* or lymphom* or myelom* or myelodysplas* or myeloproliferat* or non-hodgkin* or nonhodgkin* or no hodgkin* or no-hodgkin* or lymphom*<br>#15 (burkitt) NEAR/1 (lymphom* or neoplasm* or cancer* or tumor*)<br>#16 #1 or #2 or #3 or #4 or #5 or #6 or #7 or #8 or #9 or #10 or #11 or #12 or #13 or #14 or #15<br>#17 MeSH descriptor: [Hematopoietic Stem Cell Transplantation] explode all trees<br>#18 MeSH descriptor: [Bone Marrow Transplantation] explode all trees<br>#19 MeSH descriptor: [Transplantation, Autologous] explode all trees<br>#20 MeSH descriptor: [Peripheral Blood Stem Cell Transplantation] explode all trees<br>#21 ASCT or HSCT or autotra?splant* or auto-tra*splant* or ((autolog*) NEAR/4 (transplant* or trasplant* or rescu*))<br>#22 #17 or #18 or #19 or #20 or #21<br>#23 MeSH descriptor: [Education] explode all trees<br>#24 MeSH descriptor: [Patient Education as Topic] explode all trees<br>#25 MeSH descriptor: [Patient Education Handout] explode all trees<br>#26 MeSH descriptor: [Education, Nonprofessional] explode all trees<br>#27 MeSH descriptor: [Consumer Health Information] explode all trees<br>#28 MeSH descriptor: [Health Educators] explode all trees<br>#29 MeSH descriptor: [Teaching Materials] explode all trees<br>#30 MeSH descriptor: [Teaching] explode all trees<br>#31 MeSH descriptor: [Learning] explode all trees<br>#32 MeSH descriptor: [Programmed Instructions as Topic] explode all trees<br>#33 (patient* or caregiver* or consumer*) NEAR/1 (educat* or informat* or literacy or training* or counsel* or instruction* or advice*)<br>#34 (informati* service*) or (informati* dissemination) or (consumer health information*) or educat* or teaching or training or learning or instruction* or counsel?ing<br>#35 (therapeutic or nursing or program* or intervention* or training*) NEAR/1 (Educat*)<br>#36 (teaching or training or learning) NEAR/1 (material*)<br>#37 (health) NEAR/1 (education or information or literacy or program*)<br>#38 #23 or #24 or #25 or #26 or #27 or #28 or #29 or #30 or #31 or #32 or #33 or #34 or #35 or #36 or #37<br>#39 #16 and #22 and #38 in Trials	455
