# Supplementary material for: Nursing Educational Interventions for Oncohaematological Patients Undergoing Autologous Stem Cell Transplantation and Their Caregivers: A Mixed-Methods Systematic Review
Source: Healthcare (Basel). 2026 Nov 1;14(21):3693. doi: 10.3390/healthcare14213693 (PMC13763544; doi:10.3390/healthcare14213693)
Supplement: Supplementary file 1 [file healthcare-14-03693-s001.zip › Supplementary Table S2. MMAT appraisal criteria and supporting explanations.pdf]

Supplementary Table S2. MMAT appraisal criteria and supporting explanations

Study Design: Quantitative non-randomized

1. Wang et al., 2023 [46]

**S1**	Are there clear research questions?	Yes
**S2**	Do the collected data allow to address the research questions?	Yes
**1**	Are the participants representative of the target population?	Yes
**2**	Are measurements appropriate regarding both the outcome and intervention (or exposure)?	Yes
**3**	Are there complete outcome data?	Can't tell[a]
**4**	Are the confounders accounted for in the design and analysis?	Can't tell[b]
**5**	During the study period, is the intervention administered (or exposure occurred) as intended?	Yes

[a] *No information is provided regarding dropouts or missing data in the questionnaires.*

[b] *Although the authors reported using ANCOVA to adjust for potential confounders, adjusted effect estimates and confidence intervals were not provided. Inconsistencies in the reported outcome data further limited the assessment of the adjusted analysis.*

2. Wu et al., 2025 [48]

**S1**	Are there clear research questions?	Yes
**S2**	Do the collected data allow to address the research questions?	Yes
**1**	Are the participants representative of the target population?	Can't tell[c]
**2**	Are measurements appropriate regarding both the outcome and intervention (or exposure)?	Yes
**3**	Are there complete outcome data?	Can't tell[d]
**4**	Are the confounders accounted for in the design and analysis?	No[e]
**5**	During the study period, is the intervention administered (or exposure occurred) as intended?	Can't tell[f]

[c] *Although eligibility criteria were specified, the retrospective design and insufficient reporting of patient selection limit the assessment of sample representativeness.*

[d] *The completeness of outcome data and the management of missing data were not adequately reported.*

[e] *Potential confounders were not accounted for in the analysis.*

[f] *The consistency of intervention delivery could not be established, as detailed implementation monitoring was not reported.*

Study Design: Quantitative descriptive

1. Mank & Molenaar, 2008 [47]

**S1**	Are there clear research questions?	Yes
**S2**	Do the collected data allow to address the research questions?	Yes
**1**	Is the sampling strategy relevant to address the research question?	Yes
**2**	Is the sample representative of the target population?	Can't tell[g]
**3**	Are the measurements appropriate?	Can't tell[h]
**4**	Is the risk of nonresponse bias low?	No[i]
**5**	Is the statistical analysis appropriate to answer the research question?	Yes

[g] *Although consecutive patients were recruited, 18% did not complete the questionnaire, including patients who were too ill or emotionally distressed to use the CD-ROM. This may have affected the representativeness of the final sample.*

[h] *The study-specific questionnaire underwent preliminary testing with five patients, but no psychometric results documenting its validity or reliability were reported.*

[i] *Eleven of the 62 patients (18%) did not complete the questionnaire: seven were too ill or emotionally distressed to use the CD-ROM, two died, and two could not be contacted. These reasons for nonresponse suggest a potential risk of bias.*

2. Salvador, 2006 [45]

**S1**	Are there clear research questions?	Yes
**S2**	Do the collected data allow to address the research questions?	Yes
**1**	Is the sampling strategy relevant to address the research question?	Yes
**2**	Is the sample representative of the target population?	Can't tell[l]
**3**	Are the measurements appropriate?	Can't tell[m]
**4**	Is the risk of nonresponse bias low?	Yes
**5**	Is the statistical analysis appropriate to answer the research question?	Yes

[l] *Small sample size (n=10).*

[m] *The study did not provide sufficient information on the measurement procedures or the validity and reliability of the assessment methods used.*

Study Design: Qualitative Study

1. Halpin & Konomos, 2022 [42]

**S1**	Are there clear research questions?	Yes
**S2**	Do the collected data allow to address the research questions?	Yes
**1**	Is the qualitative approach appropriate to answer the research question?	Yes
**2**	Are the qualitative data collection methods adequate to address the research question?	Yes
**3**	Are the findings adequately derived from the data?	Yes
**4**	Is the interpretation of results sufficiently substantiated by data?	Yes
**5**	Is there coherence between qualitative data sources, collection, analysis and interpretation?	Yes

2. Halpin, 2026 [43]

**S1**	Are there clear research questions?	Yes
**S2**	Do the collected data allow to address the research questions?	Yes
**1**	Is the qualitative approach appropriate to answer the research question?	Yes
**2**	Are the qualitative data collection methods adequate to address the research question?	Yes
**3**	Are the findings adequately derived from the data?	Yes
**4**	Is the interpretation of results sufficiently substantiated by data?	Yes
**5**	Is there coherence between qualitative data sources, collection, analysis and interpretation?	Yes

3. Tarzian et al., 1999 [44]

**S1**	Are there clear research questions?	Yes
**S2**	Do the collected data allow to address the research questions?	Yes
**1**	Is the qualitative approach appropriate to answer the research question?	Yes
**2**	Are the qualitative data collection methods adequate to address the research question?	Yes
**3**	Are the findings adequately derived from the data?	Yes
**4**	Is the interpretation of results sufficiently substantiated by data?	Yes
**5**	Is there coherence between qualitative data sources, collection, analysis and interpretation?	Yes
